# SIRT1 restoration enhances chondrocyte autophagy in osteoarthritis through PTEN-mediated EGFR ubiquitination

**DOI:** 10.1038/s41420-022-00896-8

**Published:** 2022-04-15

**Authors:** Qunshan Lu, Peilai Liu, Zhuang Miao, Desu Luo, Songlin Li, Mei Lu

**Affiliations:** 1 Department of Orthopedics, Qilu Hospital of Shandong University, Jinan, 250012 P.R. China; 2 Cheeloo College of Medicine, Shandong University, Jinan, 250100 P.R. China; 3 Department of Geriatric Medicine, Qilu Hospital of Shandong University, Jinan, 250012 P.R. China; 4 Key Laboratory of Cardiovascular Proteomics of Shandong Province, Qilu Hospital of Shandong University, Jinan, 250012 P.R. China

**Keywords:** Bone, Osteoarthritis

## Abstract

The pharmacological interventions aimed at activating pathways inducing chondrocyte autophagy or reversing extracellular matrix degradation may be promising approaches for the management of osteoarthritis (OA). Evidence exists suggesting that sirtuin 1 (SIRT1) is involved in the pathogenesis of OA. The present study aimed to explore the regulatory role and downstream mechanisms of SIRT1 in OA. Bioinformatics predictions identified downstream factors phosphatase and tensin homolog (PTEN) and epidermal growth factor receptor (EGFR) in OA. We validated poorly expressed SIRT1 and EGFR and highly expressed PTEN in cartilage tissues of OA patients. OA was induced in vitro by exposing human primary chondrocytes to IL-1β and in vivo by destabilization of the medial meniscus (DMM) in a mouse model. SIRT1 knockdown was found to augment IL-1β-stimulated inflammation and chondrocyte metabolic imbalance. Knockdown of SIRT1 diminished PTEN acetylation and then enhanced PTEN expression. PTEN inactivation decreased EGFR ubiquitination and promoted EGFR expression by destabilizing the EGFR-Cbl complex, which in turn inhibited extracellular matrix degradation in cartilage tissues and activated chondrocyte autophagy. In the DMM mouse model, knockdown of SIRT1 inhibited chondrocyte autophagy, promoted metabolic imbalance, thus accelerating osteoarthritic process. In conclusion, SIRT1 represses the ubiquitination of EGFR by down-regulating PTEN, inhibits extracellular matrix degradation and activates chondrocyte autophagy, thereby performing an OA-alleviating role.

## Introduction

Osteoarthritis (OA) is a common rheumatism characterized by cartilage rupture and synovial inflammation [1]. OA results in a decline in physical functions and life qualities in a progressive manner [2]. Besides, cartilage degeneration that participates in the development of OA may induce proinflammatory cytokines including IL-1β and stimulate inflammatory response [3]. In addition, the autophagy, which is considered as a homeostatic pathway, is decreased in OA chondrocytes and the reduction in autophagy contributes to the development of OA [4]. Due to limited knowledge with regard to the exact molecular mechanism involved in the chondrocyte autophagy and the development of OA, there is no effective way to treat OA, apart from total joint replacement [5].

Sirtuin 1 (SIRT1) is a nicotinamide adenine dinucleotide (NAD)-dependent histone deacetylase [6]. SIRT1 participates in the silymarin-based regulation on chondrocyte phenotype in OA [7]. Moreover, activated AMPK-SIRT1 signaling suppresses chondrocyte apoptosis, further contributing to OA therapy [8]. Furthermore, in the ethanol-induced myocardial injury, SIRT1 is able to mediate phosphatase and tensin homolog (PTEN), thus affecting mitochondrial apoptosis [9]. Chondrocyte proliferation and apoptosis are influenced by the miR-337-3p/PTEN axis with altered status in OA [10]. PTEN also regulates epidermal growth factor receptor (EGFR) ubiquitination, and inactivation of PTEN specifically increases EGFR activity [11]. Reduced EGFR activity accelerates cartilage degeneration [12]. Moreover, a previous study stated that mice with cartilage-specific EGFR deficiency developed accelerated knee OA [13].

The aforementioned evidence suggested that SIRT1 may has a role to confer in the modulation of OA development through a mechanism involving PTEN-mediated EGFR ubiquitination. Therefore, in this study, we explored the functional relevance of the SIRT1/PTEN/EGFR axis in the pathogenesis of OA based on the in vivo DMM mouse model and the IL-1β-induced chondrocyte-like in vitro microenvironment.

## Results

### Bioinformatic identification of downstream regulators of SIRT1 in OA

**Fig. 1:**
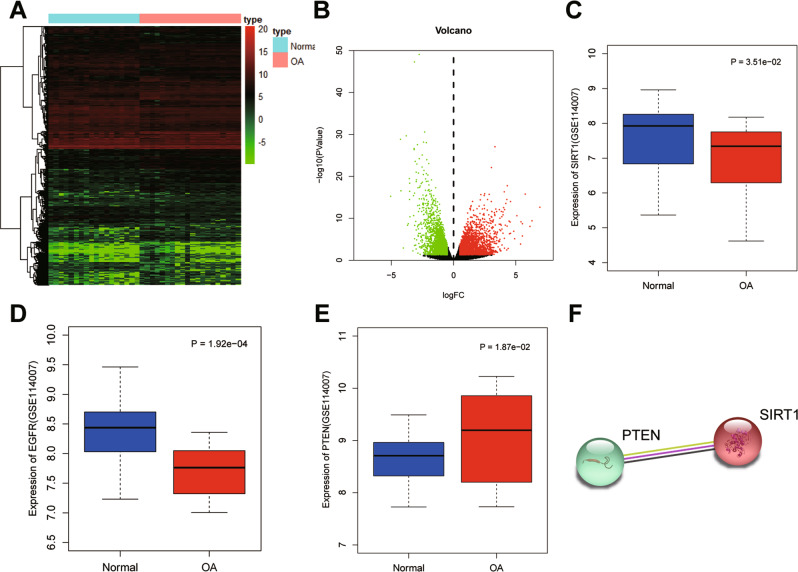
Bioinformatic identification of SIRT1-based mechanism in OA. **A** Heat map of differential gene expression screened in OA on GSE114007 dataset. *X* axis indicates the sample name, and the *Y* axis indicates the gene name. Each grid in the figure represents the expression of each gene in the corresponding sample. **B** Volcano map of differential gene expression screened in OA on GSE114007. *X* axis indicates the negative logarithm of *p* value (The green dots indicate low expression genes in OA; red dots indicate high expression genes in OA, while black dots indicate no significant difference genes). **C** Expression of SIRT1 in OA (*n* = 20) and normal (*n* = 18) samples. **D** Expression of EGFR gene in OA (*n* = 20) and normal (*n* = 18) samples. **E** PTEN expression in OA (*n* = 20) and normal (*n* = 18) samples. **F** STRING protein database analysis of protein–protein interactions of SIRT1 and PTEN.

In the OA-related microarray dataset GSE114007, 3145 high and 2487 low expressed genes were screened in OA cartilage tissue samples (Fig. 1A, B). Among them, SIRT1 and EGFR were poorly expressed in OA samples, and PTEN was highly expressed in OA samples (Fig. 1C–E). Protein–protein interaction (PPI) analysis revealed that SIRT1 interacted with PTEN protein (PPI enrichment *p* < 0.05) (Fig. 1F). PTEN has been reported to regulate EGFR ubiquitination, and inactivation of PTEN may elevate EGFR activity [11]. Besides, reduced EGFR activity might accelerate cartilage degradation [12]. The above results allowed us to suspect that the deacetylase SIRT1 may be involved in OA development through regulating PTEN-mediated EGFR.

### Underexpression of SIRT1 occurs in cartilage tissues from patients with OA

**Fig. 2:**
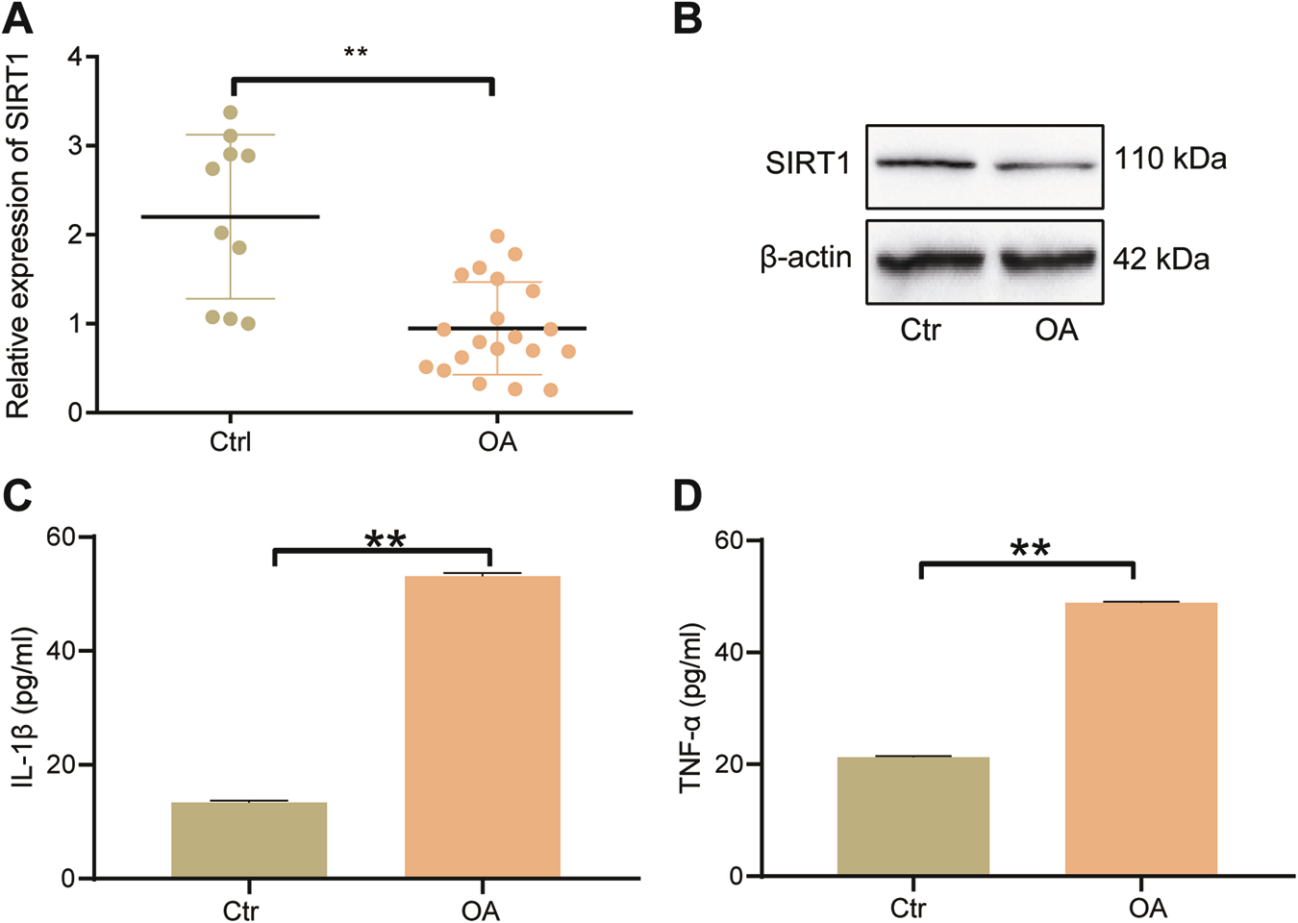
Quantification of SIRT1 expression and inflammatory factor production in patients with OA. **A** qRT-PCR to detect the expression of SIRT1 mRNA in normal cartilage tissue (*n* = 10) and OA cartilage tissue (*n* = 20). **B** Western blot to detect the expression of SIRT1 protein in normal cartilage tissue (*n* = 10) and OA cartilage tissue (*n* = 20). **C** ELISA to detect the level of IL-1β in serum of OA patients (*n* = 20). **D** ELISA to detect the level of TNF-α in serum of OA patients (*n* = 20). **p < 0.01 compared with Ctrl group.

Normal cartilage tissue and OA cartilage tissue samples were collected, where we found diminished SIRT1 mRNA expression in cartilage tissues from patients with OA versus normal cartilage tissues (Fig. 2A). Consistently, the SIRT1 protein expression in cartilage tissues from patients with OA was also downregulated (Fig. 2B). In addition, the serum levels of IL-1β and TNF-α were elevated in OA patients than normal samples (Fig. 2C, D). These results suggest that the expression of SIRT1 is down-regulated in cartilage tissues of patients with OA and may be related to inflammation.

### SIRT1 expression is decreased in IL-1β-stimulated primary human chondrocytes

There is evidence that the level of IL-1β in the blood of patients with OA is significantly higher than that of normal people [14]. The increased expression of IL-1β can cause the degradation of extracellular matrix of cartilage tissue, inhibit the synthesis of important components of extracellular matrix (Collagen II and Aggrecan) in chondrocytes, and promote the expression of MMPs [15]. Therefore, like other studies, we chose to treat primary human chondrocytes with IL-1β to simulate the OA-like microenvironment in vitro.

**Fig. 3:**
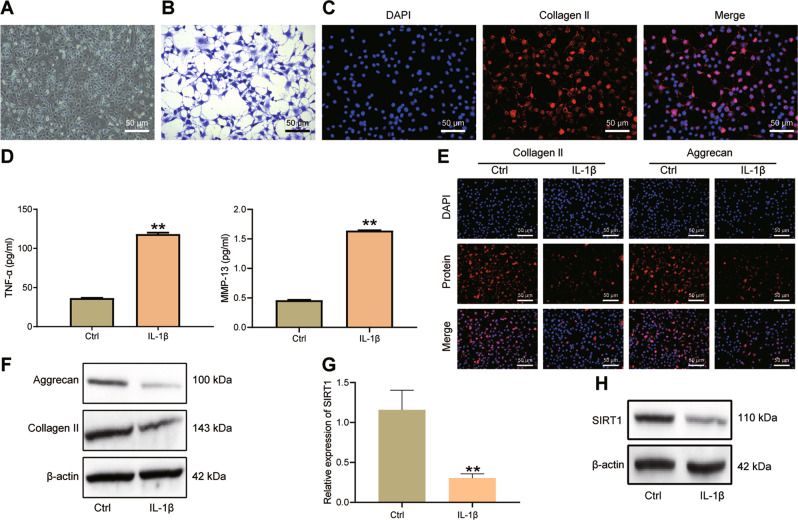
Characterization of SIRT1 expression in IL-1β-stimulated chondrocytes in vitro. **A** Microscopic image of primary human chondrocytes. **B** Toluidine blue staining to detect Proteoglacan expression in human chondrocytes in response to IL-1β stimulation. **C** Immunofluorescence staining assay to detect Collagen II expression (red fluorescence) in human chondrocytes in response to IL-1β stimulation. **D** ELISA to detect TNF-α and MMP-13 in the cell supernatant of primary human chondrocytes in response to IL-1β stimulation. **E** Immunofluorescence staining for the expression of Aggrecan and Collagen II in human chondrocytes in response to IL-1β stimulation. **F** Western blot for the protein expression of Collagen II and Aggrecan in primary human chondrocytes in response to IL-1β stimulation. **G** qRT-PCR detection of SIRT1 in primary human chondrocytes in response to IL-1β stimulation. **H** Western blot detection of SIRT1 protein expression in primary human chondrocytes in response to IL-1β stimulation. **p < 0.01 compared with Ctrl group. Cell experiments were performed in triplicates.

We first identified the extracted primary human chondrocytes (Fig. 3A). The cells extracted from the OA cartilage tissue specimens were chondrocytes, as the presence of chondrocyte markers Proteoglacan and Collagen II was validated by Toluidine blue staining and immunofluorescence staining (Fig. 3B, C).

Further, the levels of TNF-α and MMP-13 in the chondrocyte supernatant were increased after IL-1β stimulation (Fig. 3D). Meanwhile, Collagen II (red fluorescence) and Aggrecan (red fluorescence) were located in the cell cytoplasm, and no red fluorescence was seen in the nucleus (blue fluorescence). Collagen II and Aggrecan were reduced in chondrocytes after IL-1β stimulation (Fig. 3E). The expression of Collagen II and Aggrecan proteins were down-regulated after 10 ng/ml IL-1β exposure in primary human chondrocytes for 24 h (Fig. 3F). These results indicate that IL-1β-stimulated primary human chondrocytes can be used as a cellular model to simulate the OA-like microenvironment in vitro.

Moreover, our results displayed reduced mRNA and protein expression of SIRT1 in IL-1β-challenged primary human chondrocytes (Fig. 3G, H).

Collectively, down-regulation of SIRT1 was confirmed in the IL-1β-induced primary human chondrocytes.

### SIRT1 specifically increases EGFR activity by disrupting EGFR ubiquitination via PTEN

**Fig. 4:**
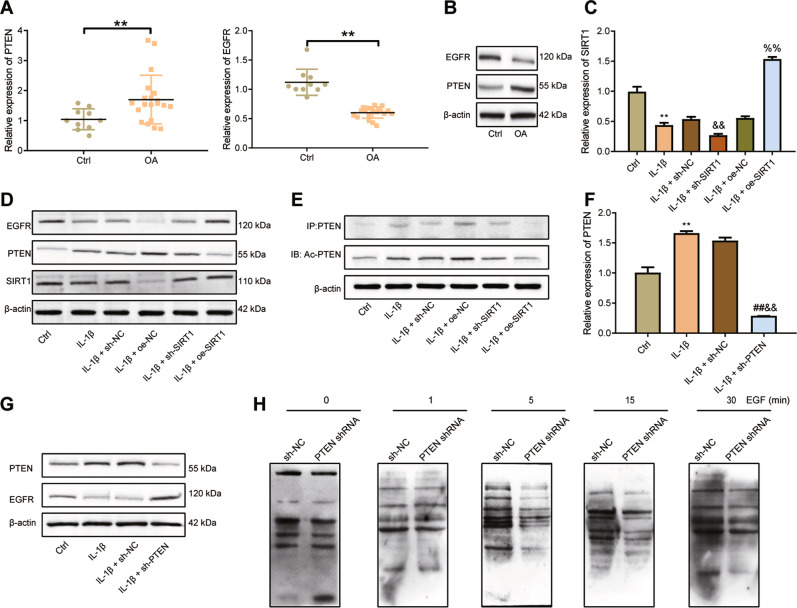
SIRT1 overexpression reduces EGFR ubiquitination by suppressing PTEN expression. **A** qRT-PCR detection of PTEN and EGFR expression in normal (*n* = 10) and OA (*n* = 20) cartilage tissues. **B** Western blot detection of PTEN and EGFR protein expression in normal (*n* = 10) and OA (*n* = 20) cartilage tissues. **C** qRT-PCR detection of SIRT1 transduction efficiency in IL-1β-challenged primary human chondrocytes. **D** Western blot detection of expression levels of SIRT1, PTEN and EGFR proteins in IL-1β-challenged primary human chondrocytes after transduction with oe-SIRT1 or sh-SIRT1. **E** Immunoprecipitation assay of acetylation of PTEN in IL-1β-challenged primary human chondrocytes after oe-SIRT1 or sh-SIRT1 treatment. **F** qRT-PCR measurement of PTEN expression in response to sh-PTEN treatment. **G** Western blot detection of PTEN and EGFR protein expression in IL-1β-challenged primary human chondrocytes after transduction with sh-PTEN. **H** Detection of EGFR ubiquitination caused by EGF-stimulated chondrocytes after knockdown of PTEN. **p < 0.01 compared with the Ctrl group. ^##^*p* < 0.01 com*p*ared with IL-1β group. ^&&^*p* < 0.01 compared with IL-1β + sh-NC group. ^%%^*p* < 0.01 compared with IL-1β + oe-NC group.

To verify whether SIRT1 acted through PTEN to regulate EGFR, we first examined the expression of PTEN and EGFR in clinical cartilage tissue samples. PTEN expression was elevated and EGFR expression was lowered in cartilage tissues of OA patients compared with normal cartilage tissues (Fig. 4A). PTEN protein expression was upregulated and EGFR protein expression was decreased in cartilage tissues of OA patients (Fig. 4B).

To further investigate the regulatory mechanism of SIRT1 on PTEN and EGFR, we knocked SIRT1 down or overexpressed SIRT1 in IL-1β-stimulated chondrocytes. SIRT1 mRNA and protein expression was appreciably decreased in response to sh-SIRT1 and increased in response to oe-SIRT1, indicating successful transduction of sh-SIRT1 and oe-SIRT1 in IL-1β-stimulated chondrocytes (Fig. 4C, D). Further, IL-1β exposure led to down-regulated SIRT1 and EGFR protein expression as well as up-regulated PTEN. In the IL-1β-challenged chondrocytes, PTEN protein expression was elevated in the presence of sh-SIRT1 and diminished in the presence of oe-SIRT1, while EGFR protein expression was decreased upon sh-SIRT1 transduction and elevated upon oe-SIRT1 transduction (Fig. 4D). As shown in immunoprecipitation using an antibody toward PTEN (Fig. 4E), exposure to IL-1β resulted in an increase in the Ac-PTEN protein expression in chondrocytes, which were magnified upon sh-SIRT1 transduction yet reversed in response to oe-SIRT1 transduction.

Meanwhile, PTEN mRNA expression was reduced after transduction of sh-PTEN, which confirmed successful PTEN knockdown (Fig. 4F). PTEN protein expression was also decreased, and EGFR protein expression was up-regulated in the presence of sh-PTEN (Fig. 4G). As depicted in detection of EGFR ubiquitination (Fig. 4H), epidermal growth factor (EGF) stimulation for 5 min led to EGFR ubiquitination, and EGF-induced EGFR ubiquitination in chondrocytes was not changed upon PTEN knockdown.

Based on the aforementioned results, it is shown that knockdown of SIRT1 diminished PTEN acetylation and then enhanced PTEN expression, and that SIRT1 restoration-induced inactivation of PTEN specifically enhanced EGFR activity by disrupting EGFR ubiquitination.

### SIRT1 restoration upregulates EGFR expression and alleviates chondrocyte metabolic imbalance in OA via PTEN

**Fig. 5:**
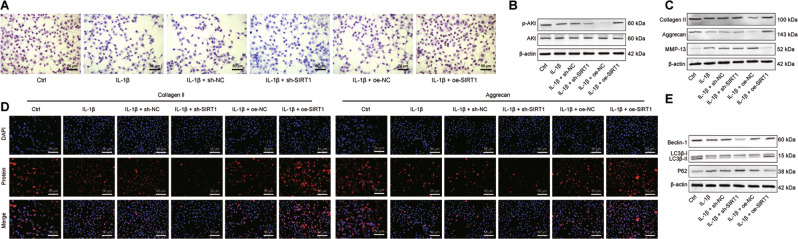
SIRT1 affects chondrocyte synthesis, metabolic imbalance and autophagy in OA by upregulating EGFR via PTEN. **A** Safranin O staining to detect the deposition of GAGs in human chondrocytes in the presence of SIRT1 knockdown or restoration. **B** Western blot to detect p-Akt and Akt protein expression levels in human chondrocytes in the presence of SIRT1 knockdown or restoration. **C** Western blot for Collagen II, Aggrecan and MMP-13 in human chondrocytes in the presence of SIRT1 knockdown or restoration. **D** Immunofluorescence staining assay for Collagen II (green fluorescence) and Aggrecan (red fluorescence) after knockdown or restoration of SIRT1. **E** Western blot assay to detect the expression levels of Beclin-1, LC3B-I, LC3B-II and p62 proteins in human chondrocytes after knockdown or restoration of SIRT1. Cell experiments were performed in triplicates.

Using safranin O staining, it was found that the deposition of glycosaminoglycans (GAGs) was restricted in IL-1β-stimulated chondrocytes relative to control chondrocytes, and additional transduction of sh-SIRT1 further diminished the GAG deposition yet oe-SIRT1 transduction reversed the deposition, suggesting a protective effect of SIRT1 restoration in the OA cell model (Fig. 5A). The IL-1β induction alone led to decreases in Akt phosphorylation and p-Akt/Akt ratio in chondrocytes, and its combination with sh-SIRT1 transduction enlarged the decreases whereas oe-SIRT1 transduction abrogated the decreases, indicating that the Akt pathway was activated after SIRT1 restoration and played a protective role against OA (Fig. 5B).

Moreover, IL-1β exposure resulted in reduced expression of chondrocyte synthesis markers (CollagenII and Aggrecan) expression and elevated expression of the chondrocyte lysis marker MMP-13; additional sh-SIRT1 transduction further downregulated CollagenII and Aggrecan expression and upregulated MMP-13, thus aggravating the metabolic imbalance in cartilage tissue. Meanwhile, the combination with oe-SIRT1 transduction, relative to IL-1β stimulation alone, manifested upregulated CollagenII and Aggrecan and downregulated MMP-13 expression (Fig. 5C). Consistent results were obtained in immunofluorescence staining (Fig. 5D), confirming that SIRT1 restoration alleviated the metabolic imbalance induced by IL-1β in chondrocytes.

Further to determine whether SIRT1 has a role to confer in chondrocyte autophagy after IL-1β treatment, we used Western blot to assay the effect of SIRT1 on autophagy markers Beclin-1, LC3B and p62. Decreases in Beclin-1 expression and LC3B-II/LC3B-I ratio and an increase in p62 expression were observed in chondrocytes after exposure to IL-1β, and these IL-1β-induced variations were augmented in response to additional knockdown of SIRT1 yet negated in the presence of SIRT1 restoration (Fig. 5E). Altogether, these experimental data evidenced that the abnormally diminished expression of SIRT1 contributed to cartilage degeneration in OA cartilage tissue and also the IL-1β-mediated chondrocyte autophagy by downregulating EGFR, thus subsequently accelerated the OA progression.

### SIRT1 blunts the progression of OA in mice through modulation of PTEN/EGFR

**Fig. 6:**
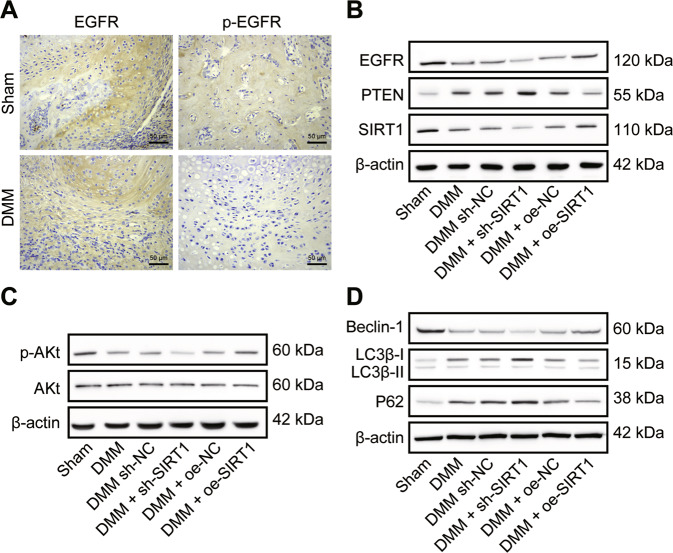
SIRT1 regulates the PTEN/EGFR axis and alleviates the osteoarthritic process in DMM mice. **A** Immunohistochemical detection of EGFR and p-EGFR expression in cartilage tissue of DMM mice. **B** Western blot detection of SIRT1, PTEN and EGFR protein expression levels in DMM mice after transduction of sh-SIRT1 or oe-SIRT1. **C** Western blot detection of p-Akt and Akt protein expression levels in DMM mice after transduction of sh-SIRT1 or oe-SIRT1. **D** Western blot detection of Beclin-1, LC3B-I, LC3B-II and p62 protein expression levels in DMM mice after knockdown of SIRT1 and transduction with SIRT1 (*n* = 10 mice per group).

To investigate the effects of SIRT1, PTEN and EGFR on the OA model in vivo, we established a DMM mouse model combined with sh-SIRT1 or oe-SIRT1 interventions. Immunohistochemical analysis identified EGFR-positive staining in all articular chondrocytes. DMM modeling induced a phenotype mimicking OA in the knee joint of mice. Although no significant morphological changes occurred in articular cartilage morphology and EGFR-positive chondrocytes, p-EGFR-positive chondrocytes disappeared from the articular cartilage surface (Fig. 6A). This suggested a successful modeling.

Further, SIRT1 and EGFR protein levels were down-regulated and the PTEN protein level was up-regulated in the DMM mice, and additional SIRT1 restoration led to a larger decrease in EGFR and a larger increase in PTEN expression while knockdown of SIRT1 reversed the changes in EGFR and PTEN levels (Fig. 6B). The p-Akt protein levels and the p-Akt/Akt ratio were lower after DMM molding. After knockdown of SIRT1, the p-Akt/Akt ratio decreased in DMM mice. After SIRT1 restoration, p-Akt and Akt protein levels were reversed in the sham-operated mice, and the p-Akt/Akt ratio was increased (Fig. 6C).

Beclin-1 protein expression was downregulated, p62 protein expression was upregulated, and LC3B-II/LC3B-I was downregulated after DMM modeling. Knockdown of SIRT1 resulted in down-regulation of Beclin-1 protein expression, up-regulation of p62 protein expression, and a decrease in LC3B-II/LC3B-I ratio in DMM mice. SIRT1 restoration resulted in the reversal of Beclin-1, LC3B-II and p62 protein levels in DMM mice (Fig. 6D).

Taken together, the results showed that knockdown of SIRT1 affected the extracellular matrix, matrix degrading enzyme content, and cellular autophagy of cartilage tissue in vivo, leading to accelerated development of OA; and these impacts could be mitigated by SIRT1 restoration.

### Perturbation of the SIRT1/PTEN/EGFR axis regulates OA progression in vivo

**Fig. 7:**
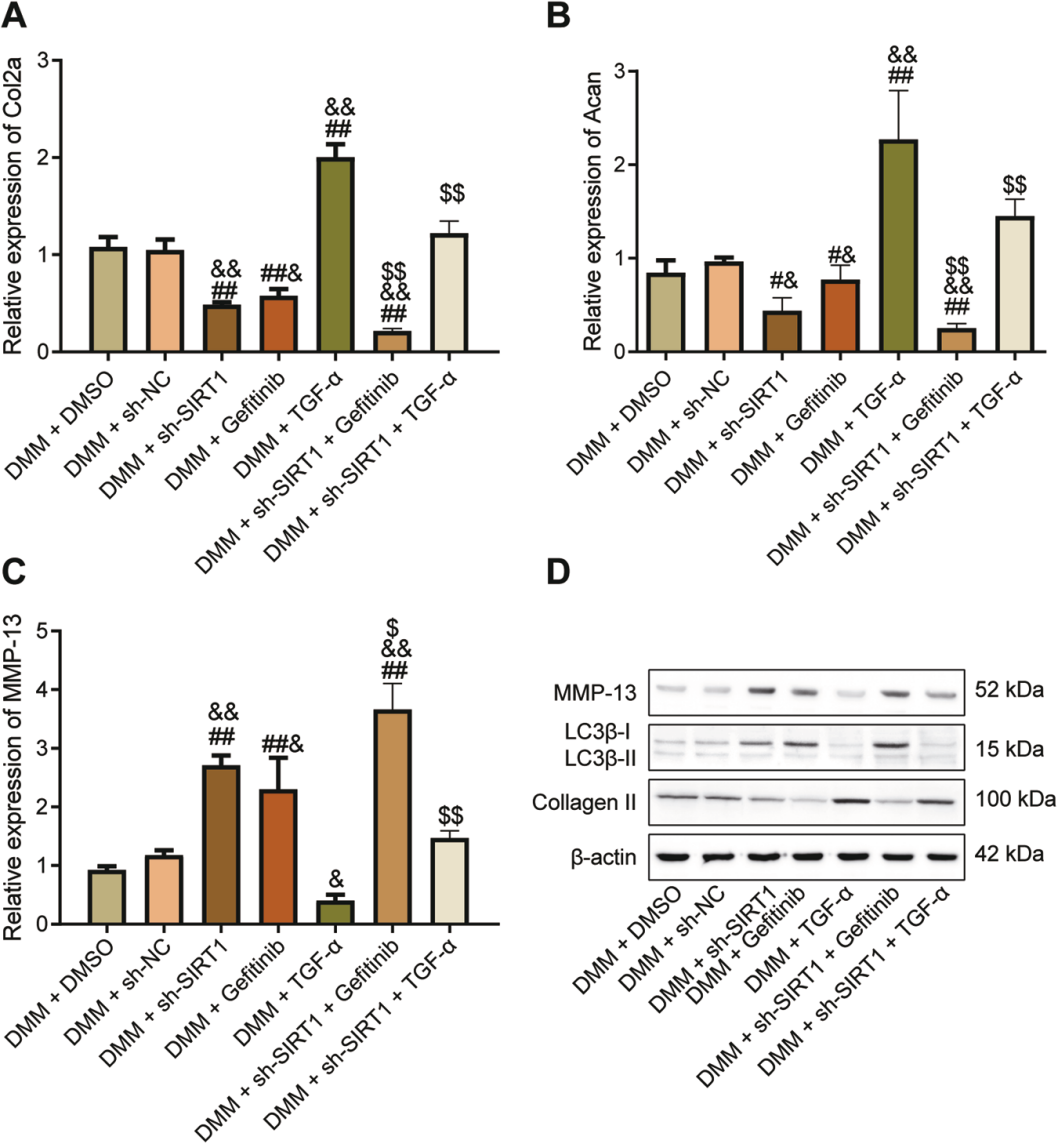
Alteration of SIRT1/PTEN/EGFR axis regulates the process of OA in vivo. **A** qRT-PCR detection of Col2a gene expression in mouse chondrocytes in response to TGF-α and gefitinib. **B** qRT-PCR detection of Acan gene expression in mouse chondrocytes in response to TGF-α and gefitinib. **C** qRT-PCR detection of MMP-13 gene expression in mouse chondrocytes in response to TGF-α and gefitinib. **D** Western blot detection of MMP-13, LC3B-I, LC3B-II and Collagen protein expression in chondrocytes from mice with TGF-α and gefitinib interventions. ^#^*p* < 0.05, ^##^*p* < 0.01 com*p*ared with the DMM + DMSO group. ^&^*p* < 0.05, ^&&^*p* < 0.01 compared with the DMM + sh-NC group. ^$^*p* < 0.05, ^$$^*p* < 0.01 compared with DMM + sh-SIRT1 group.

To further validate our results, we stimulated joints of DMM mice with TGF-α (EGFR activator) and gefitinib (EGFR inhibitor), respectively. Gefitinib and sh-SIRT1 inhibited Col2a and Acan expression and increased MMP-13 levels, while TGF-α increased Col2a and Acan expression but decreased MMP-13 levels. Gefitinib synergistically enhanced the inhibitory effect of sh-SIRT1 on Col2a and Acan and the elevated effect of MMP-13, while TGF-α hindered the OA-promoting effect of sh-SIRT1 (Fig. 7A–C). Gefitinib synergistically enhanced the OA-promoting effect of sh-SIRT1, while TGF-α impeded the OA-promoting effect of sh-SIRT1, mainly by upregulating Collagen II expression and LC3B-II/LC3B-I ratio and downregulating MMP-13 protein expression (Fig. 7D).

Therefore, SIRT1 plays a protective role against OA by inhibiting extracellular matrix degradation in OA cartilage tissue through PTEN-mediated suppression of EGFR ubiquitination, promoting synthesis of extracellular matrix, and activating chondrocyte autophagy.

## Discussion

OA treatment requires early proactive management but currently early-stage diagnostic criteria are unavailable [16]. Among many treatments, intra-articular delivery of biologics against molecules or cells shows great promise, including biologic therapies, cell therapies and growth factor therapy [17]. Here, we defined the protective effect of SIRT1 restoration against OA progression in enhancing chondrocyte autophagy and suppressing extracellular matrix degradation.

Our bioinformatics analysis results predicted the underexpression of SIRT1 and EGFR as well as overexpression of PTEN in OA, which were further verified in clinical samples and in animal and cell models of OA. A previous study in a rat model of OA indicated abnormal SIRT1 loss in chondrocytes and the curcumin-induced SIRT1 elevation displayed appreciable alleviatory effect on OA [18]. SIRT1 exerts chondroprotective functions in OA, indicating that its activation holds potential in reducing cartilage degeneration [19]. Our experimental data also corroborated that SIRT1 restoration also relieved metabolic imbalance, extracellular matrix degradation and augmented chondrocyte autophagy in OA-like mice or microenvironment. Consistent evidences have reported that SIRT1 overexpression led to decreased apoptotic rates of chondrocytes [20], and SIRT1 knockdown may result in chondrocyte senescence [21]. It has been found that improved extracellular matrix degradation of IL-1β-induced chondrocytes can be led by downregulated MMP-13 and ADAMTS-5 [22]. Moreover, SIRT1 overexpression increased the collagen II and aggrecan and reduced the collagen I, collagen X, MMP-13 [23].

Mechanistic actions investigations of this study presented that knockdown of SIRT1 diminished PTEN acetylation and then enhanced PTEN expression, while PTEN inactivation decreased EGFR ubiquitination and promoted EGFR expression by destabilizing the EGFR-Cbl complex. PTEN expression has been found to be increased in OA mice [24]. Also PTEN regulates chondrocyte apoptosis and extracellular matrix degradation in interaction with miR-20b in OA [25]. Meanwhile, it has been reported that SIRT1 can interact with PTEN to directly regulate PTEN expression, and downregulation of SIRT1 leads to increased PTEN expression [26], while PTEN can regulate EGFR ubiquitination, and inactivation of PTEN can specifically increase EGFR activity [11].

Recent studies have revealed that EGFR signaling pathways are multifunctional cytokines that are closely associated with pathological and physiological processes in a variety of human organs and tissues and play an important role in regulating the growth and metabolism of chondrocytes [27]. Furthermore, EGFR signaling is critical for preventing OA initiation and maintaining the superficial layer of articular cartilage [12]. It was reported that EGFR ubiquitination is mediated by the Cbl family of RING domain E3 ubiquitin ligases, which bind to activated receptors and assemble multi-protein complexes, including hepatocyte growth factor-regulated substrates (Hrs) and other members of the endosomal sorting complex required for transport (ESCRT), which highly indicated that PTEN regulates EGFR expression through ubiquitination modifications [28]. These documents further suggest that the deacetylase SIRT1 may influence OA development through PTEN-mediated EGFR ubiquitination. More importantly, the results derived from IL-1β-induced chondrocytes were in line with the results from DMM-induced OA mouse cartilage tissues, in which SIRT1 alleviated cartilage tissue damage and cartilage matrix degradation in OA mice.

**Fig. 8:**
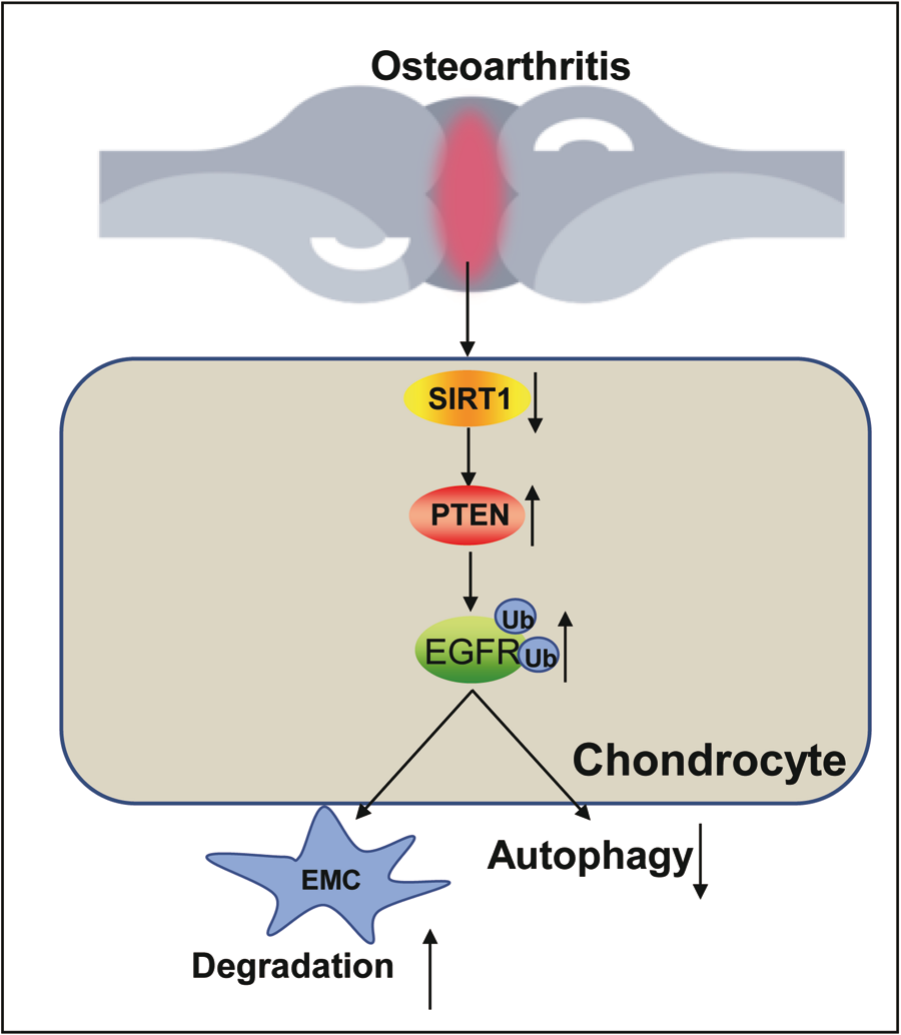
The mechanism graph of the regulatory network and function of SIRT1. SIRT1 inhibits the ubiquitination of EGFR by down-regulating PTEN, thereby inhibiting the degradation of extracellular matrix cartilage and activating chondrocyte autophagy, thereby conferring a protective role against OA. EMC, extracellular matrix.

Based on the above findings, our study proposed that SIRT1 upregulated the expression of EGFR via downregulating PTEN to hinder the development of OA (Fig. 8). However, clinical trials are still required to further clarify the mechanisms. Nevertheless, this study lays a theoretical foundation for understanding the mechanism of OA and finding new therapeutic targets.

## Materials and methods

### Ethics statement

This study was approved by the Ethics Committee of Qilu Hospital of Shandong University and written informed consent of the patients or caregivers were obtained. All animal experiments were performed under a protocol approved by the Laboratory Animal Care and Use Committee of Qilu Hospital of Shandong University.

### Bioinformatics analysis

The raw data of the OA-related microarray dataset GSE114007 was downloaded from the GEO database. The dataset consisted of 20 OA cartilage tissue samples and 18 normal tissue samples. With |log2 (fold change)| >0.5 and *p* < 0.05 as the threshold criteria, the “edgeR” package of R language was used to screen the differentially expressed genes. The expression of SIRT1, EGFR and PTEN genes in OA and normal samples was then mapped by the R language “boxplot” package. Finally, PPI analysis was performed for SIRT1 and the downstream regulator PTEN using the STRING database.

### Clinical samples

Patients with OA (aged 60–75 years, 20 cases in total) referred to Qilu Hospital of Shandong University from January 2017 to January 2018 were selected for this study. The OA cartilage tissue specimens were the remaining OA cartilage tissues except those for routine clinical examinations, which were obtained from patients who had undergone knee replacement surgery for primary knee OA. They had no history of knee surgery or rheumatoid disease, and the surface of the articular cartilage observed during the surgery was uneven, fibrotic, and cracked. Normal cartilage tissue specimens (10 cases in total) were the remaining normal cartilage tissues of the femoral head except those for routine clinical examinations, which were obtained from patients who underwent hip replacement surgery due to femoral neck fractures. They had no history of hip joint pain, hip joint surgery or rheumatoid disease, and the articular cartilage surface observed during the surgery was smooth [29]. The serum of the corresponding cases was also harvested and frozen for testing.

### Isolation and culture of primary human chondrocytes

The cartilage samples were washed with phosphate buffer saline (PBS) to remove attached blood and fat. The cartilage tissue was cut into blocks (1 × 1 × 1 cm^3^). The supernatant was discarded after centrifugation at 1200 rpm for 5 min. The trypsinized cartilage tissues were triturated and subjected to incubation with mixture containing 0.2% type II collagenase, 10% FBS, DMEM/F-12 and 1% penicillin-streptomycin at 37 °C for 24 h. The obtained precipitate after centrifugation was primary human chondrocytes, which were resuspended with culture medium containing 10% fetal bovine serum (FBS), DMEM/F12 and 1% penicillin-streptomycin and cultured at a constant temperature of 37 °C and 5% CO_2_. Cells were passaged and digested by 0.05% trypsin at 37 °C. After trituration, the cells were centrifuged, resuspended and cultured at a ratio of 1:3.

### Establishment of OA cell model

Primary human chondrocytes were trypsinized upon reaching logarithmic phase, and cells were seeded in 6-well plates at 1 × 10^5^ per well. After 24 h of conventional culture, when cell confluence reached about 75%, cells were transduced according to Lipofectamine 2000 protocols (11668019, Invitrogen, Carlsbad, CA). Cells were transduced with short hairpin RNA (shRNA) against SIRT1 (sh-SIRT1), sh-PTEN, SIRT1 overexpression vector (oe-SIRT1) and corresponding negative control (NC, sh-NC or oe-NC). After 48 h transduction, the transduction efficiency of sh-SIRT1 and sh-PTEN was detected by reverse transcription quantitative polymerase chain reaction (qRT-PCR). After 48 h transduction of primary human chondrocytes, the cell culture medium was changed to a medium with 0.5% FBS, DMEM/F12, and 1% penicillin-streptomycin in a 5% CO_2_ cell culture incubator for 1 h. Finally, 10 ng/mL IL-1β was added to the medium to mimic OA-like microenvironment, and cells transduced with various constructs were referred to as IL-1β + sh-NC, IL-1β + sh-SIRT1, IL-1β + oe-NC and IL-1β + oe-SIRT1 groups. Cells were placed at a constant temperature of 37 °C and 5% CO_2_ for an additional 24-h incubation.

### Establishment of DMM mouse model

The experimental animals used were 8-week-old C57/BL6 male mice (*n* = 100) purchased from Vital River Laboratories (Beijing, China), and were housed with 3 mice per cage with free access to food and water. OA was induced in vivo through the dissociation of the medial meniscus (DMM). For the mice in sham operation (*n* = 10), the joint capsule of the mice was incised, the meniscus was not treated, and the joint capsule was sutured, and the rest of the operation was the same as that for the DMN modeling. The mice were treated with LV5-GFP (lentiviral gene overexpression vector) or pSIH1-H1-copGFP (lentiviral shRNA fluorescent expression vector) to construct the gene lentiviral vectors, which were synthesized by GenePharma (Shanghai, China). DMM mice (10 mice per group) were untreated or treated with sh-NC, sh-SIRT1, oe-NC, or oe-SIRT1; or treated with TGF-α (EGFR activator) and gefitinib (gefitinib, EGFR inhibitor) respectively in the presence/absence of sh-SIRT1. Each mouse was injected with 40 μL of corresponding lentivirus (titer of 1 × 10^9^ TU/mL) prepared in saline to 0.1 mL. The sham-operated and untreated DMM mice were given 0.1 mL of saline.

### qRT-PCR

Total RNA was extracted from tissue cells using Trizol (10296010, Invitrogen, CA). RNA concentration, purity and integrity were determined using Nano-Drop ND-1000 spectrophotometry and 1% agarose gel electrophoresis. The RNA was reverse-transcribed into complementary DNA (cDNA) according to the instructions of PrimeScript RT reagent Kit (RR047A, Takara, Japan), and then the synthesized cDNA was analyzed by Fast SYBR Green PCR kit (Applied biosystems) with ABI PRISM 7300 RT-PCR system (Applied biosystems). The relative expression of target genes was calculated by the relative quantification method (2-∆∆Ct method) using GAPDH as the internal reference (Supplementary Table 1).

### Western blot

Cells were collected by trypsin digestion and lysed with enhanced RIPA lysis solution containing protease inhibitors (Boster, Wuhan, China), and then protein concentration was determined by BCA protein quantification kit (Boster). The proteins were separated by 5–10% sodium dodecyl sulfate polyacrylamide gel electrophoresis (SDS-PAGE), and the separated proteins were electrotransferred to PVDF membranes. Membrane was blocked by 5% BSA at room temperature for 2 h to block non-specific binding. Diluted primary antibodies of anti-SIRT1 (1:1000, ab110304, Abcam, Cambridge, UK), anti-PTEN (1:1000, ab32199, Abcam), anti-Aggrecan (1:1000, ab36861, Abcam), anti-Collagen II (1:1000, ab34712, Abcam), anti-MMP-13 (1:1000, ab39012, Abcam), anti-Beclin (1:1000, ab62557, Abcam), anti-LC3B (1:1000, ab192890, Abcam), anti-p62 (1:1000, ab109012, Abcam), anti-β-actin (1:500, ab8226, Abcam), anti-phosphorated (p)-Akt (1:1000, 9271, Cell Signaling Technology, Santa Cruz, CA), anti-Akt (1:500, 9272, Cell Signaling Technology), and anti-EGFR (1:1000, ab52894, Abcam) were incubated with the membrane overnight at 4 °C. All antibodies were rabbit-derived. The membrane was washed with HRP-labeled goat anti-rabbit secondary antibody (ab205719; 1:2000; Abcam) for 1 h at room temperature. ECL working solution (EMD Millipore) was incubated with the membrane. The grayscale quantification of bands in the Western blot images was performed using Image J analysis software, and β-actin was used as an internal reference.

### Immunoprecipitation

The protein concentration was determined by cellular protein extraction, ensuring that the amount of protein was not less than 1 mg. A small amount of lysate was taken as input for Western blot analysis, and 1 μg of the corresponding target protein antibody anti-EGFR (1:20, ab52894, Abcam), anti-PTEN (1:500, 9559, Cell Signaling Technology) and 80 μL of ProteinA/proteinG magnetic beads were incubated overnight at 4 °C with slow shaking. After the immunoprecipitation, the magnetic beads were centrifuged to the bottom of the tube, the supernatant was aspirated and discarded. Cells were eluted with 3 × SDS loading buffer. The lysis buffer contained 50 mM heparin (pH 7.4), 150 nM NaCl, 1 mM EDTA, 1 mM EGTA, 1% Nonidet P-40, 1% glycerol, protease and phosphatase inhibitors. After addition of lysis buffer, lysates were incubated on ice for 30 min without sonication and then removed by centrifugation. Western blot assay was applied for further detection.

### Immunofluorescence assay

Cells were fixed with 4% paraformaldehyde, and 0.5% Triton X-100 was used to permeabilize the cell membrane structure at room temperature. Cells were carefully removed from the cell culture plate with a fine syringe needle and placed on a slide. Slides were blocked at room temperature for 30 min by adding goat serum working solution (ZLI-9022, Zhongshan Goldenbridge Biotechnology, China) dropwise, followed by overnight incubation with diluted primary antibody, anti-Collagen II (ab34712, 1:100, Abcam) and anti-Aggrecan (13880-1-AP, 1:50, Invitrogen) at 4 °C. With addition of fluorescent secondary antibody (A32727, Invitrogen) diluted at a certain ratio, cells were further incubated for 1 h at 37 °C in a wet box. Then the nuclei with DAPI dropwise for 5 min against light. After addition of anti-fluorescence quencher dropwise, the slide was imaged under a fluorescence microscope.

### ELISA

Patient serum and cell supernatant were taken to detect the levels of IL-1β, TNF-α, MMP-13 and other related factors following the instructions of ELISA kits (BMS224-2, BMS249-4, BMS223HS, EHMMP-13, Invitrogen). The sample to be tested, negative control and standard (positive control) were added to a 96-well plate before addition of the primary antibody. Primary antibody working solution was added to each well and shaken for 60 min, following which =the enzyme standard working solution was added and shaken for 60 min and 100 μL of substrate was further added for 10-min incubation without exposure to light. The termination solution was added, followed by the measurement of the optical density value at 450 nm with ELISA reader.

### Toluidine blue staining

The medium in the 12-well cell culture plate was aspirated and discarded. Cells were fixed with 4% paraformaldehyde at room temperature for 15 min. A total of 1% toluidine blue staining solution was added for 2 h at room temperature. The toluidine blue staining solution was aspirated and discarded, then cells were carefully removed from the cell culture plate with a fine syringe needle and placed on a slide. Slides were air-dried, sealed with neutral gum, and the images were collected under the microscope.

### Saffron O staining

The medium in the 12-well cell culture plate was aspirated and discarded. Cells were fixed with 4% paraformaldehyde at room temperature for 15 min. The staining solution was added with saffron O working solution at room temperature for 30 min, which was then aspirated and discarded. Cell culture plate was placed in the scanner, and the macroscopic staining of each well was scanned and recorded. Cell culture plate was observed under the microscope and the microscopic staining of each well was photographed.

### Immunohistochemistry

Paraffin sections of clinical tissue specimens were taken, dewaxed to water, dehydrated in alcohol gradient, and repaired in water bath in antigen repair solution. Normal goat serum blocking solution (C-0005, Haoran Biotechnology, Shanghai, China) was added to the sections at room temperature for 20 min, followed by addition of primary antibodies of rabbit anti-human EGFR (1:100, ab52894, Abcam) and p-EGFR (1:100, ab5652, Abcam) overnight at 4 °C. Then sections were added with goat anti-rabbit immunoglobulin G (IgG) (ab6785, 1:1000, Abcam) secondary antibody. The tissues were placed at 37 °C for 20 min, and dripped with horseradish peroxidase-labeled streptavidin protein working solution (0343-10000U, Emo Biotech, Beijing, China) which was placed at 37 °C for 20 min. Sections were counterstained with DAB (ST033, Weijia Technology, Shanghai, China) for 1 min and returned blue with 1% ammonia. Sections were observed under a microscope and filmed with 5 high magnification fields randomly selected for each section and 100 cells were selected in each field.

### Statistical analysis

Statistical analysis of the data in this study were analyzed using SPSS 21.0 (IBM, Armonk, NY), with *p* < 0.05 as a level of statistical significance. The measurement data were expressed as mean ± standard deviation. Two independent samples were compared using unpaired *t* test. One-way analysis of variance (ANOVA) with Tukey’s post hoc test was used for comparison among multiple groups.

## Supplementary information

Supplementary Table 1

aj-checklist

uncropped western blots

## Data Availability

The datasets generated and/or analysed during the current study are available in the manuscript and Supplementary Materials.
