# Supplementary material for: SIRT1 restoration enhances chondrocyte autophagy in osteoarthritis through PTEN-mediated EGFR ubiquitination
Source: Cell Death Discov. 2022 Apr 15;8:203. doi: 10.1038/s41420-022-00896-8 (PMC9012846; doi:10.1038/s41420-022-00896-8)
Supplement: Supplementary file 1 — Supplementary Table 1 [file 41420_2022_896_MOESM1_ESM.docx]

**Supplementary table 1** Primer sequences for qRT-PCR

| Genes | Sequences (5’-3’) |
| --- | --- |
| SIRT1 (human) | Forward: AATTCCAGCCATCTCTCTGT |
|  | Reverse: CTCCTCGTACAGCTTCACAA |
| PTEN (human) | Forward: CGAACTGGTGTAATGATATGT |
|  | Reverse: CATGAACTTGTCTTCCCGT |
| EGFR (human) | Forward: GTTAAAATTCCCGTCGCTATCAAG |
|  | Reverse: TCACGTAGGCTTCATCGAGGATTTC |
| GAPDH (human) | Forward: GAAGGTGAAGGTCGGAGT |
|  | Reverse: GAGGTCAATGAAGGGGTCA |
| Col2a1 (mouse) | Forward: TCCCAGAACATCACCTACCA |
|  | Reverse: ATTGGAGCCCTGGATG |
| Acan (mouse) | Forward: GCAATTACCAGCTGCCCTTC |
|  | Reverse: TCTTCTGCCCGAGGGTTCTA |
| MMP-13 (mouse) | Forward: GATGACCTGTCTGAGGAAGACC |
|  | Reverse: GCATTTCTCGGAGCCTGTCAAC |
| GAPDH (mouse) | Forward: AATGGTGAAGGTCGGTGT |
|  | Reverse: GAGGTCAATGAAGGGGTCG |

Note: SIRT1, sirtuin 1; PTEN, phosphatase and tensin homolog; EGFR, epidermal growth factor receptor; MMP-13, matrix metalloproteinase 13; Col2a1; collagen type II alpha 1 chain; GAPDH, glyceraldehyde-3-phosphate dehydrogenase
