# Supplementary material for: SIRT1 restoration enhances chondrocyte autophagy in osteoarthritis through PTEN-mediated EGFR ubiquitination
Source: Cell Death Discov. 2022 Apr 15;8:203. doi: 10.1038/s41420-022-00896-8 (PMC9012846; doi:10.1038/s41420-022-00896-8)

Figure 2B


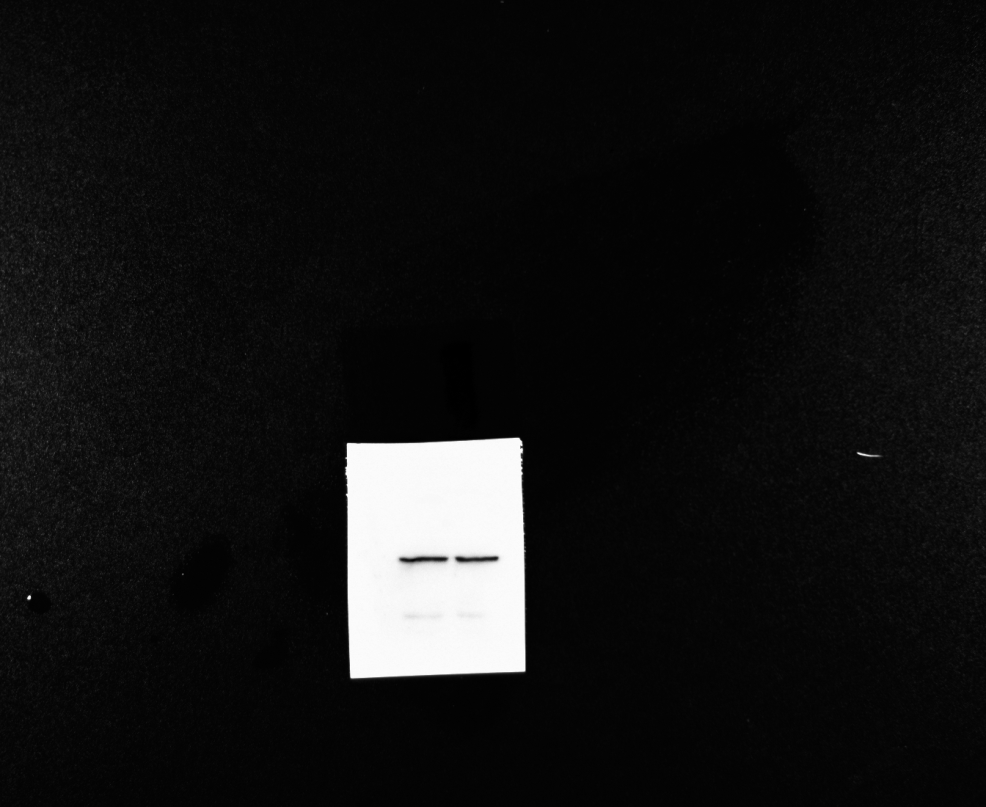

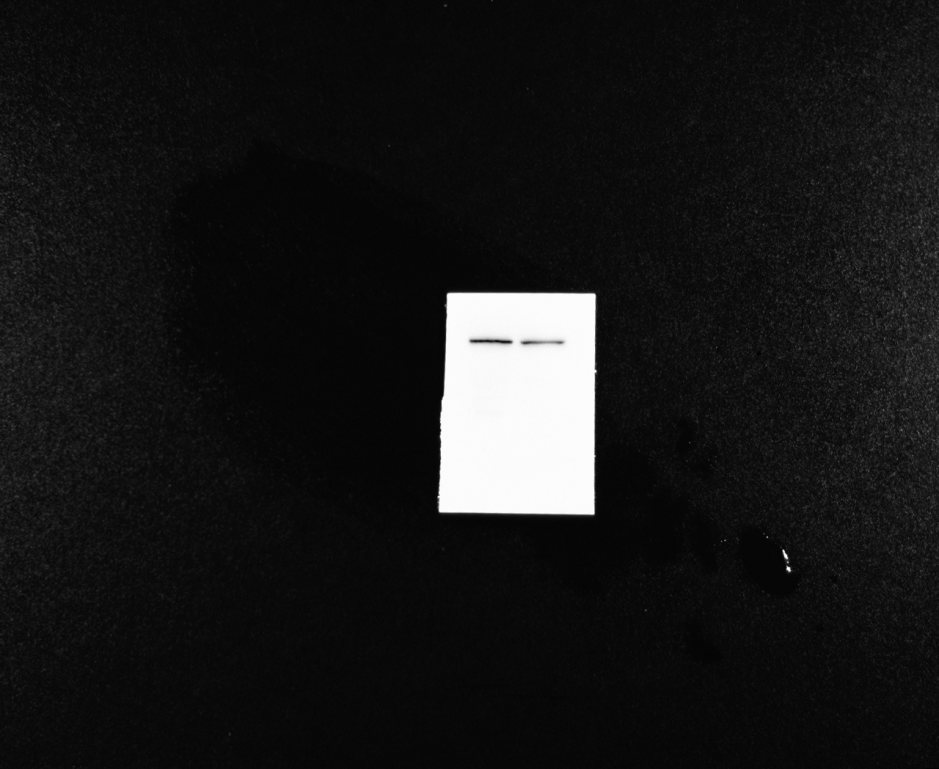


Figure 3F


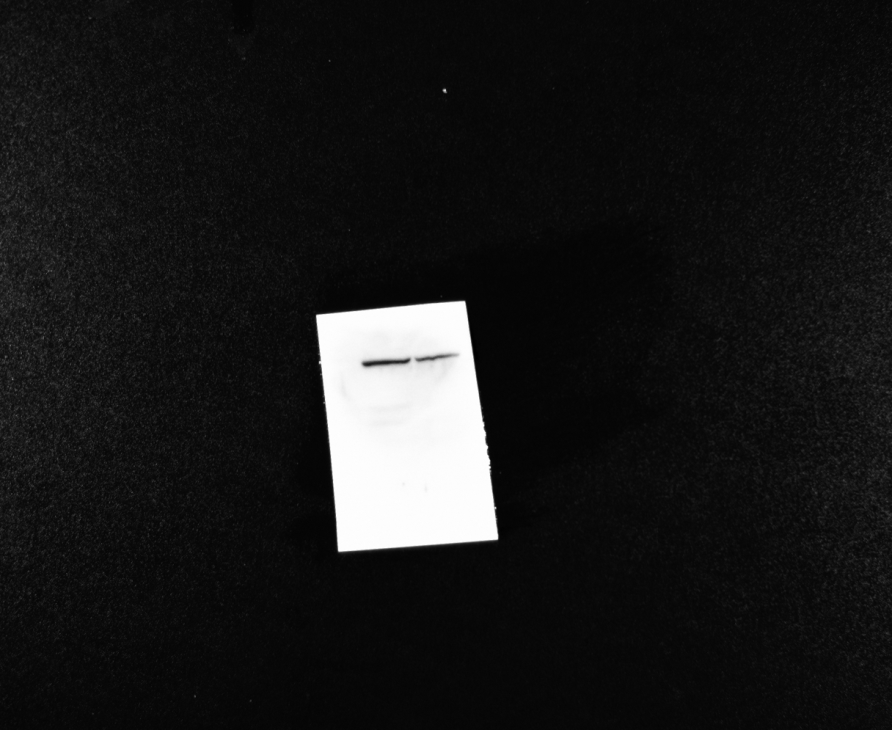

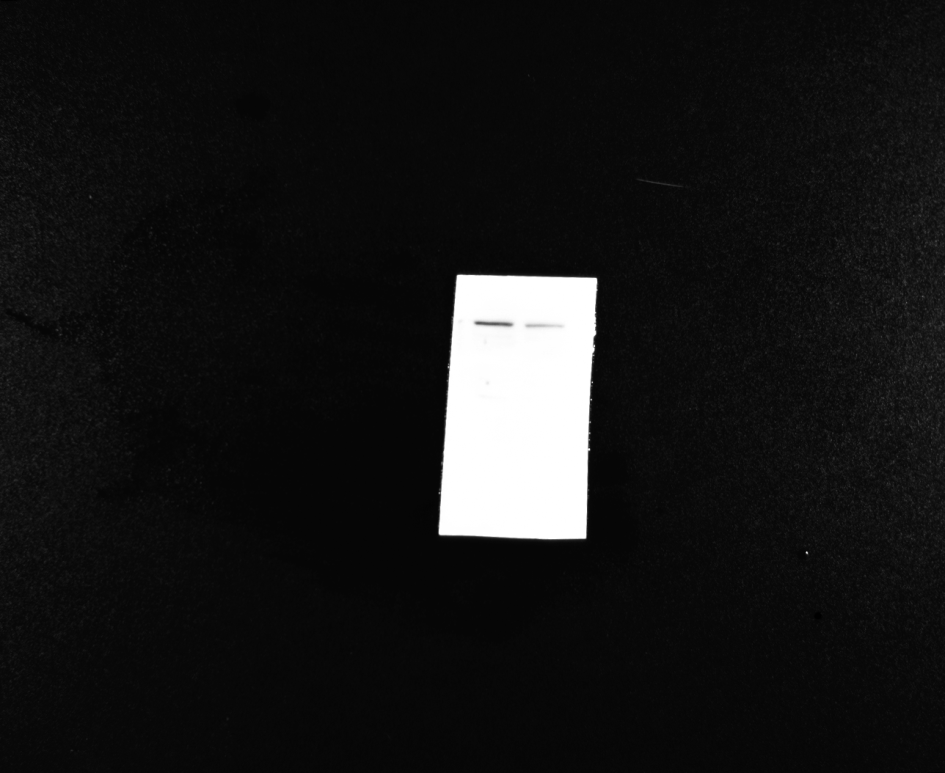

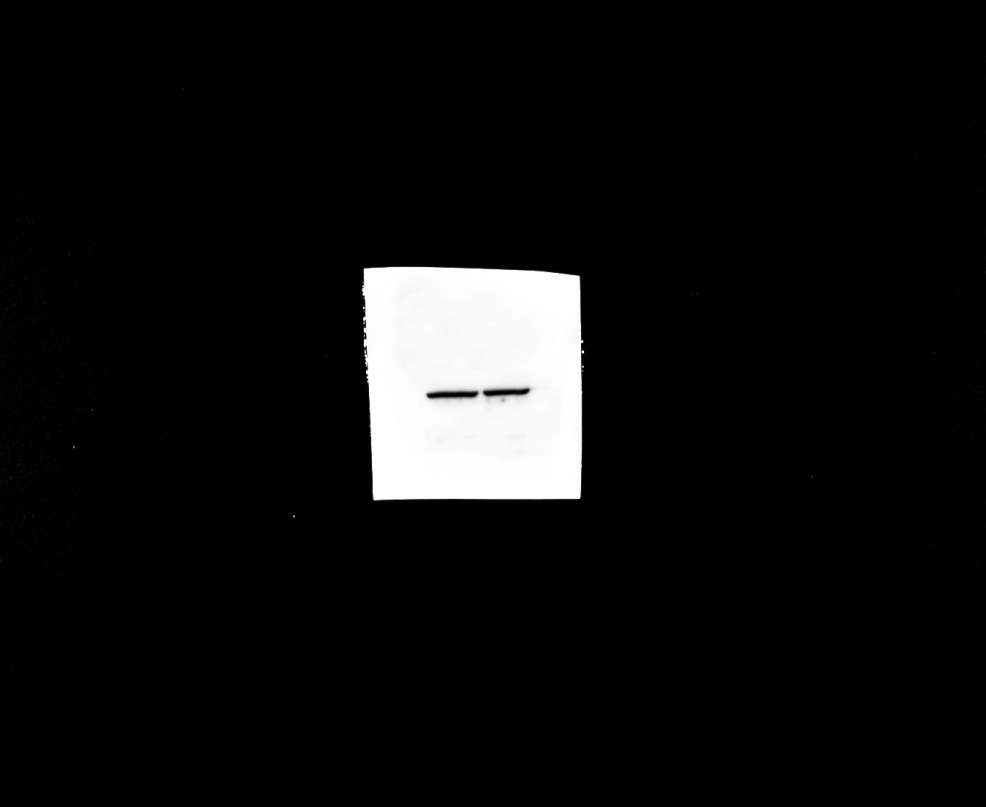


Figure 3H


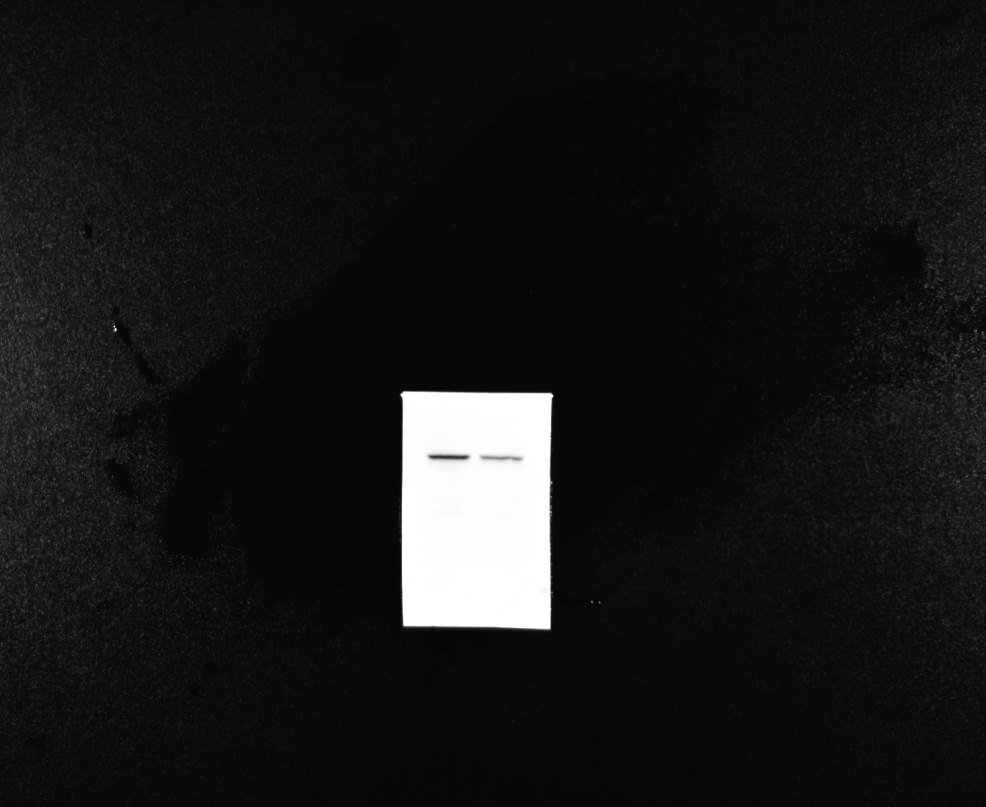

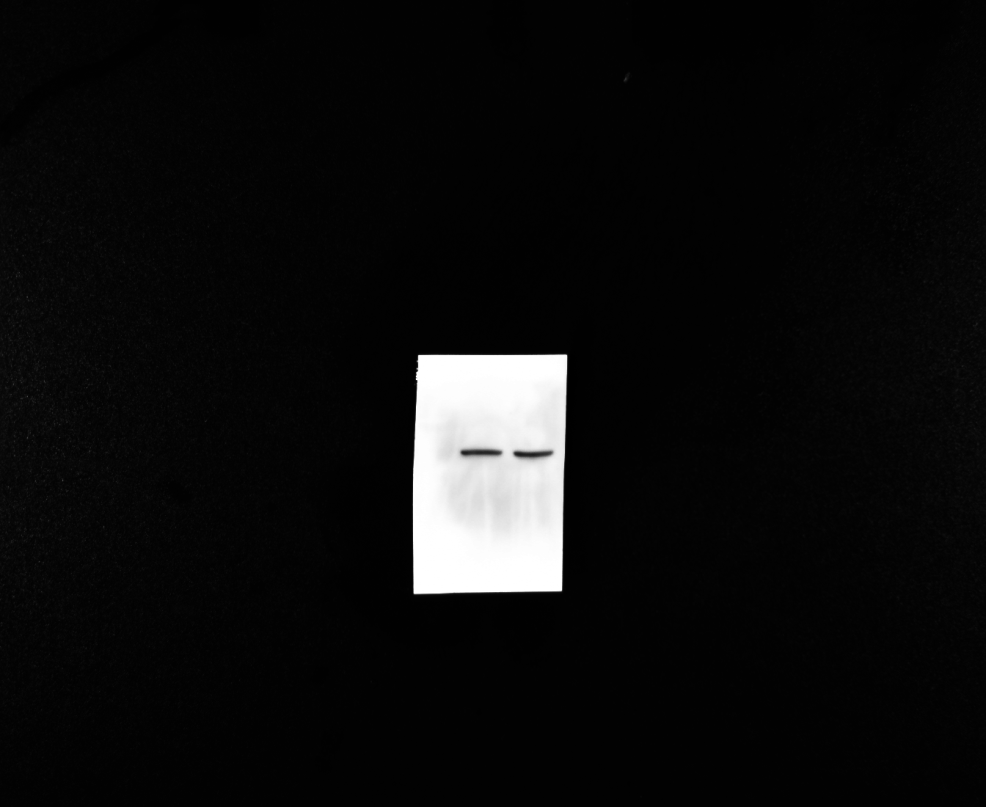


Figure 4D


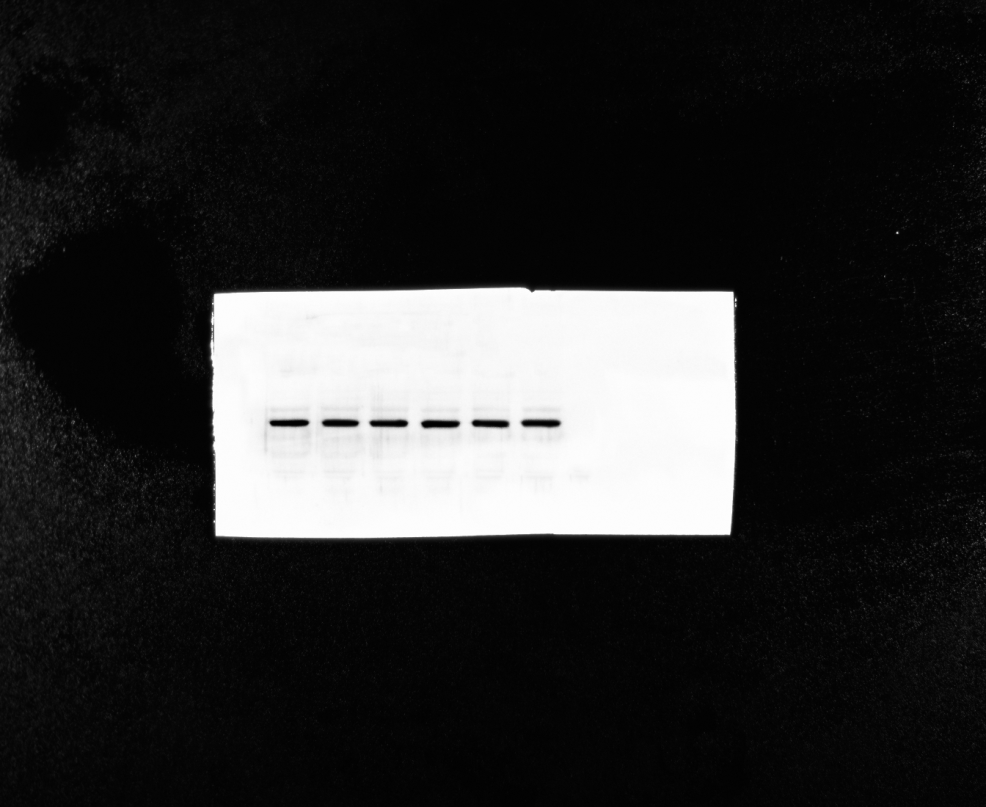

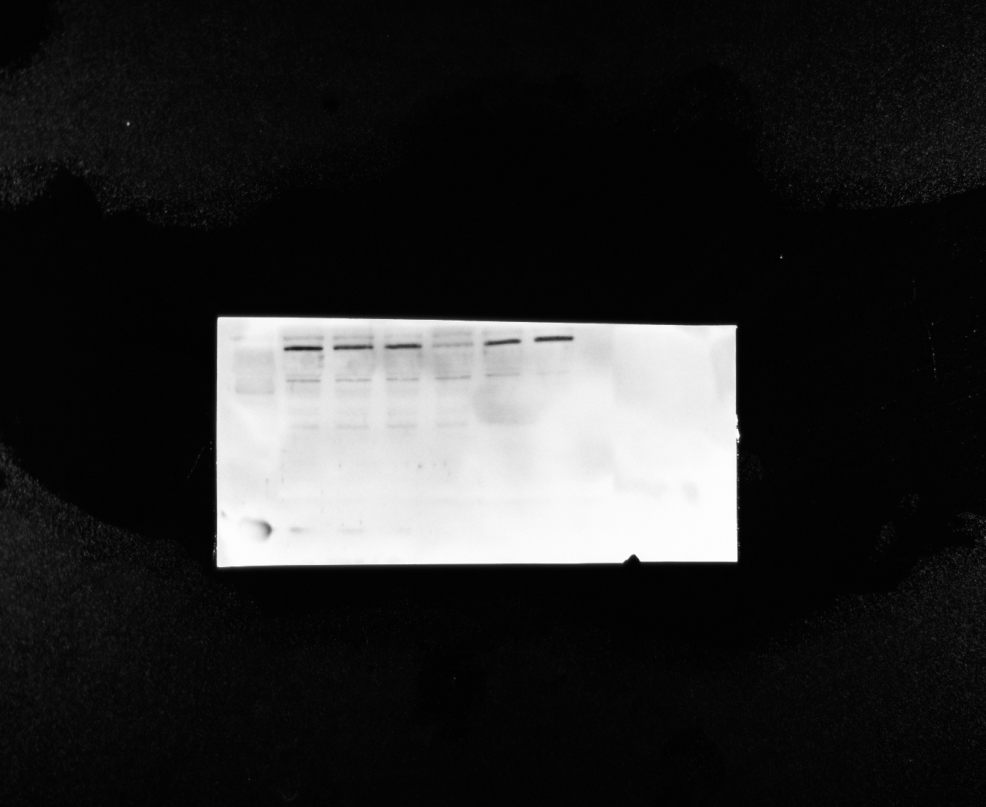

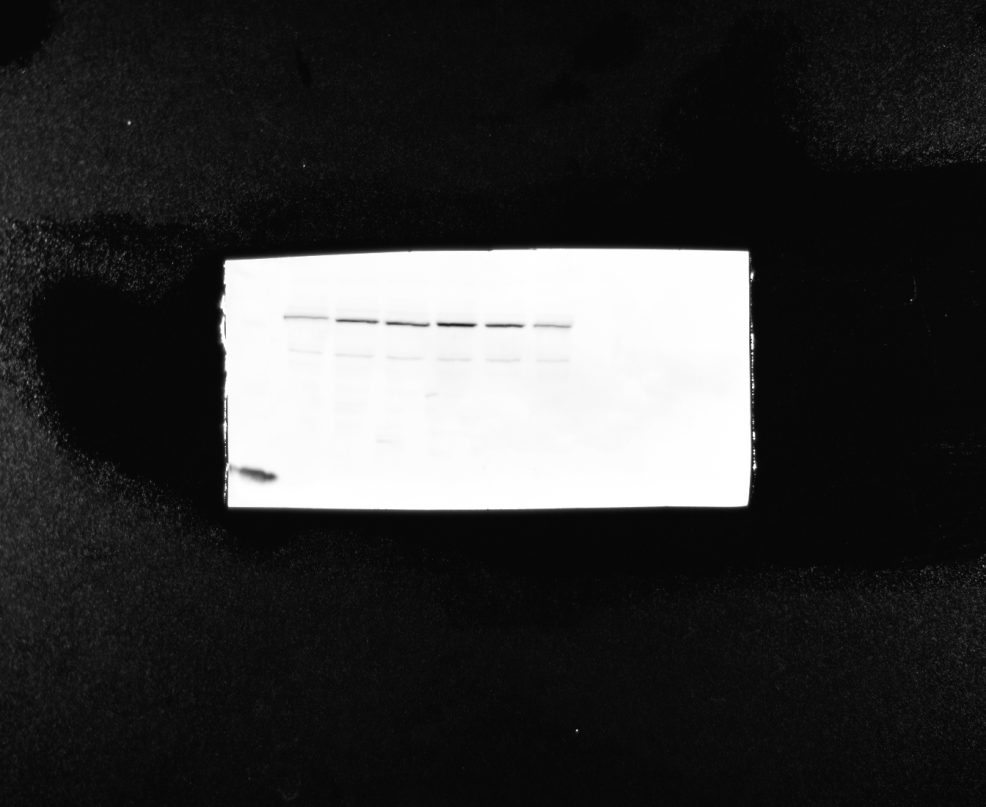

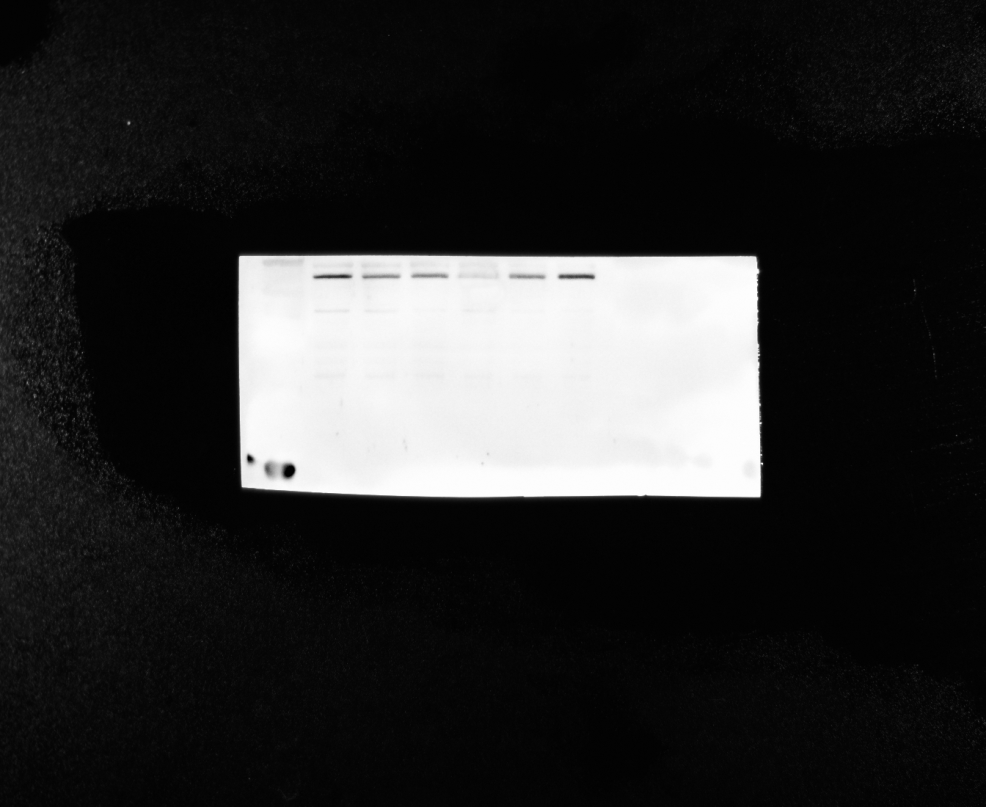


Figure 4E


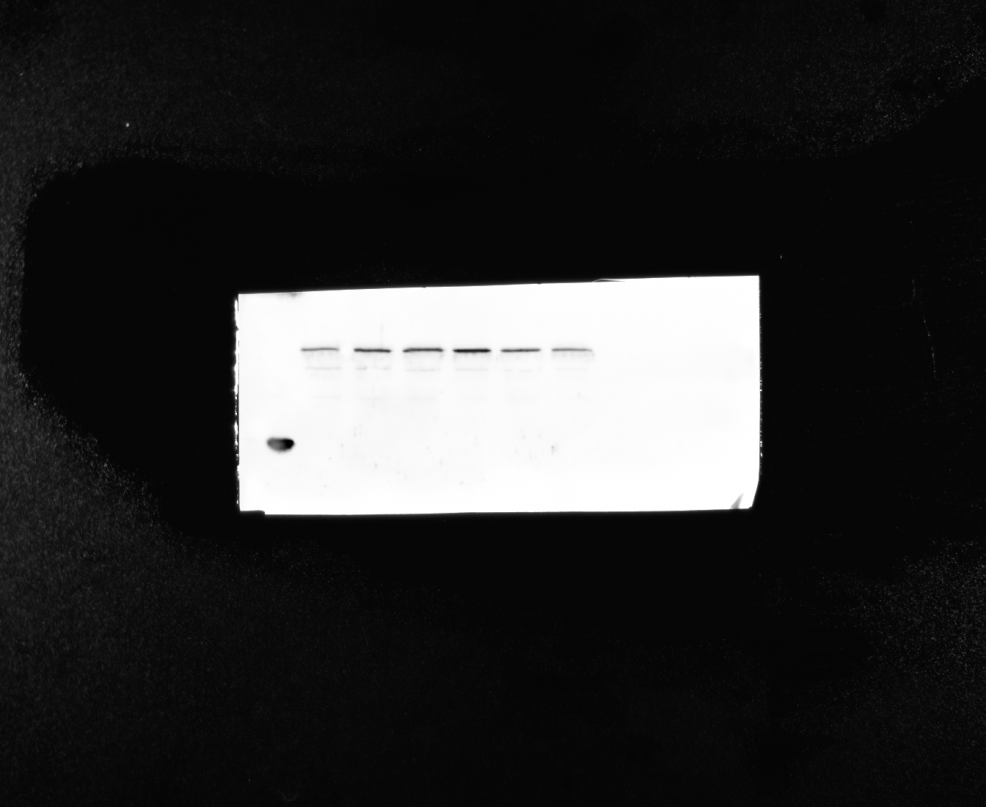

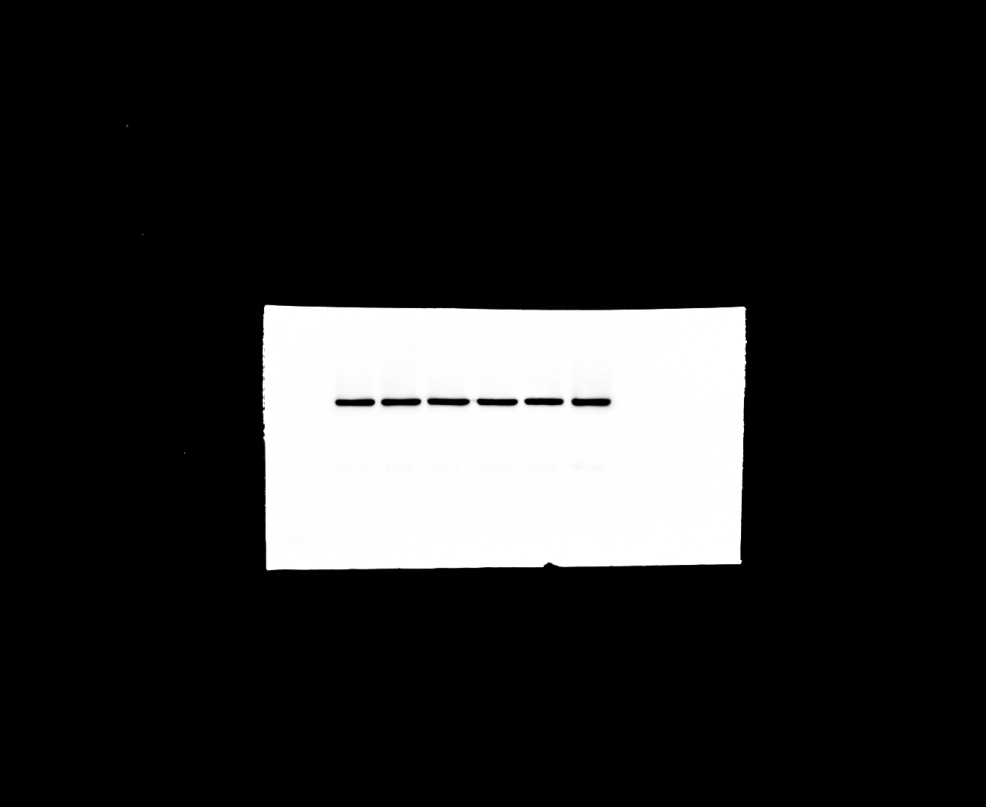

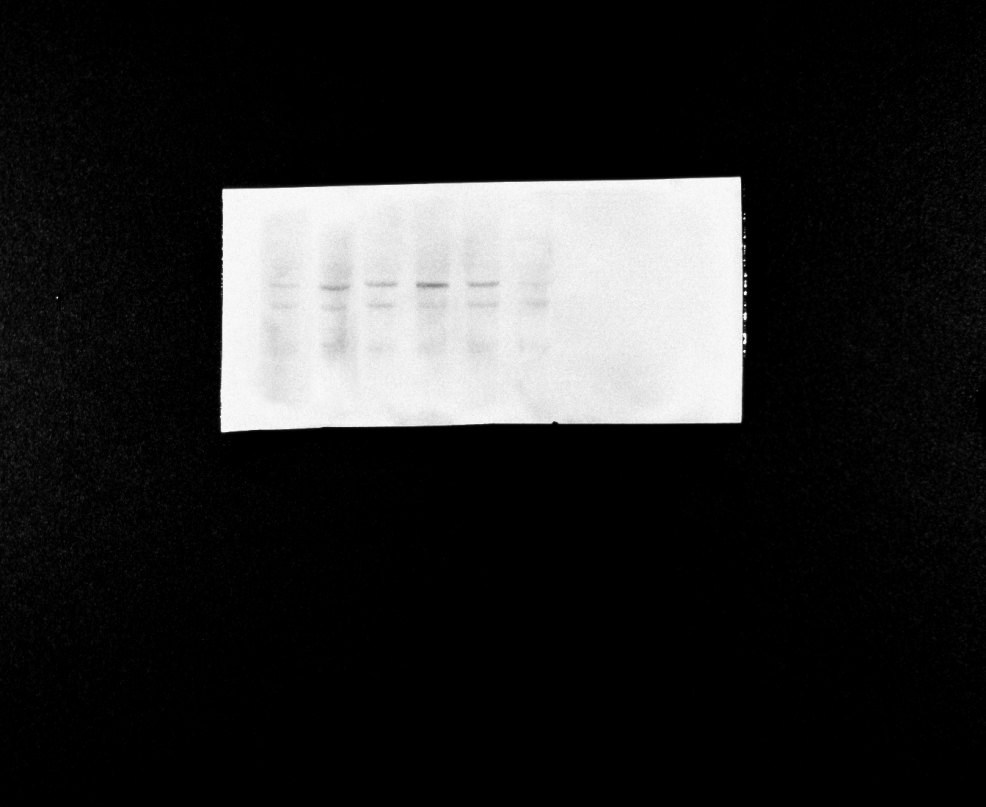


Figure 4G


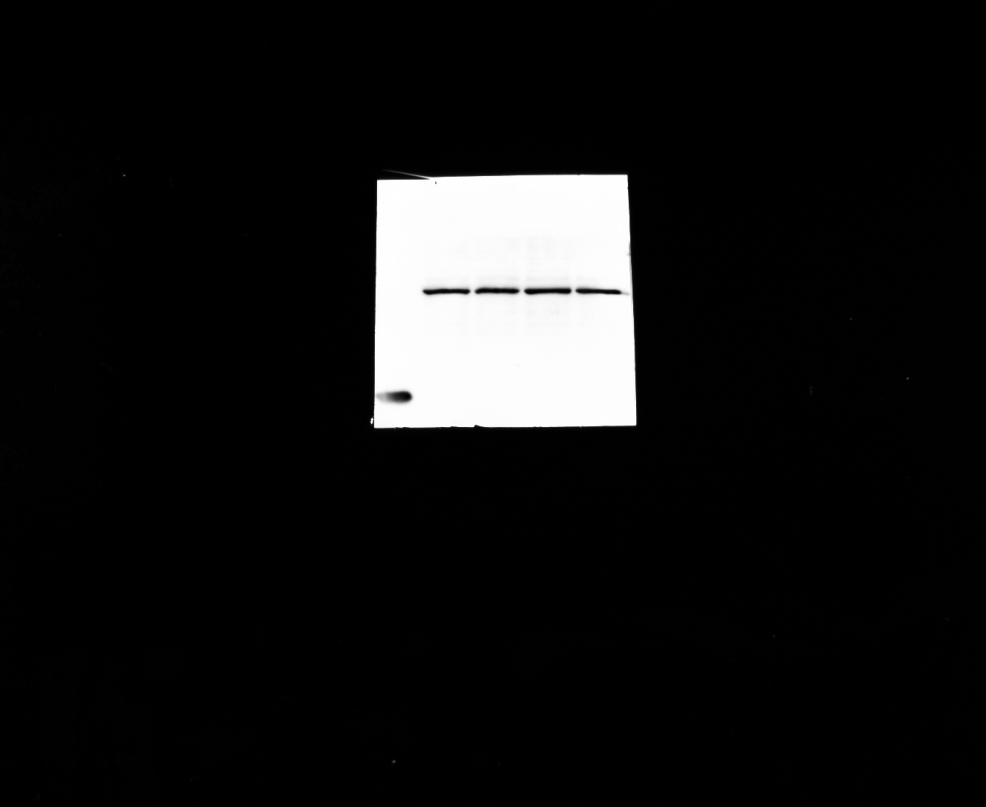

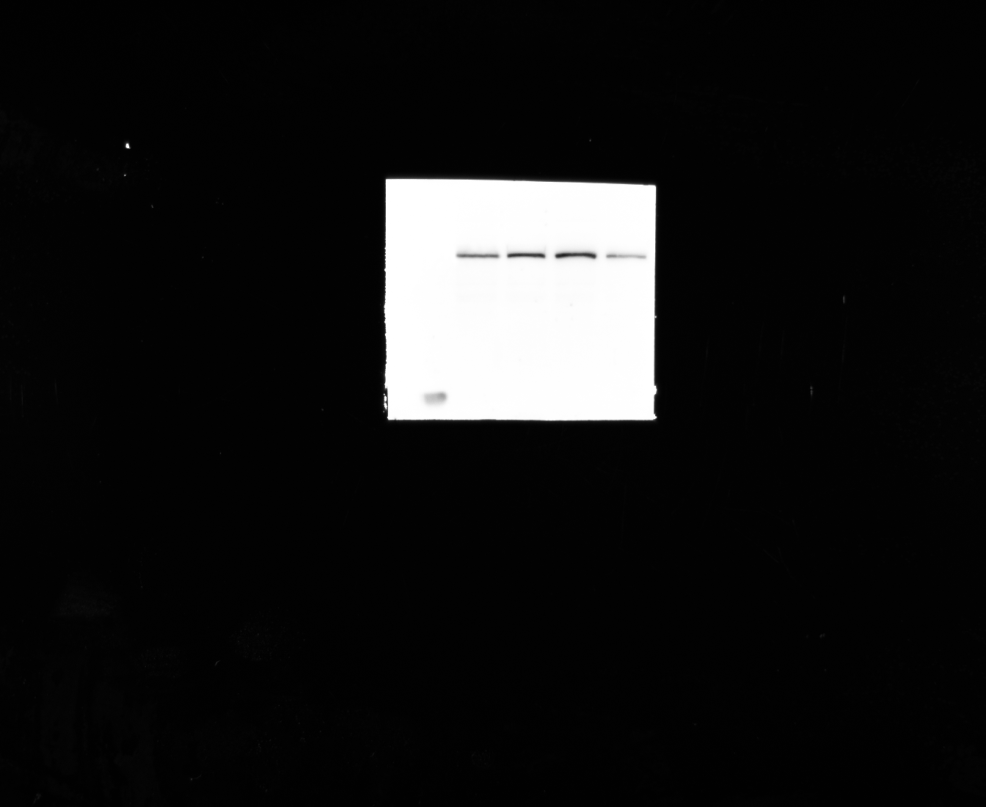

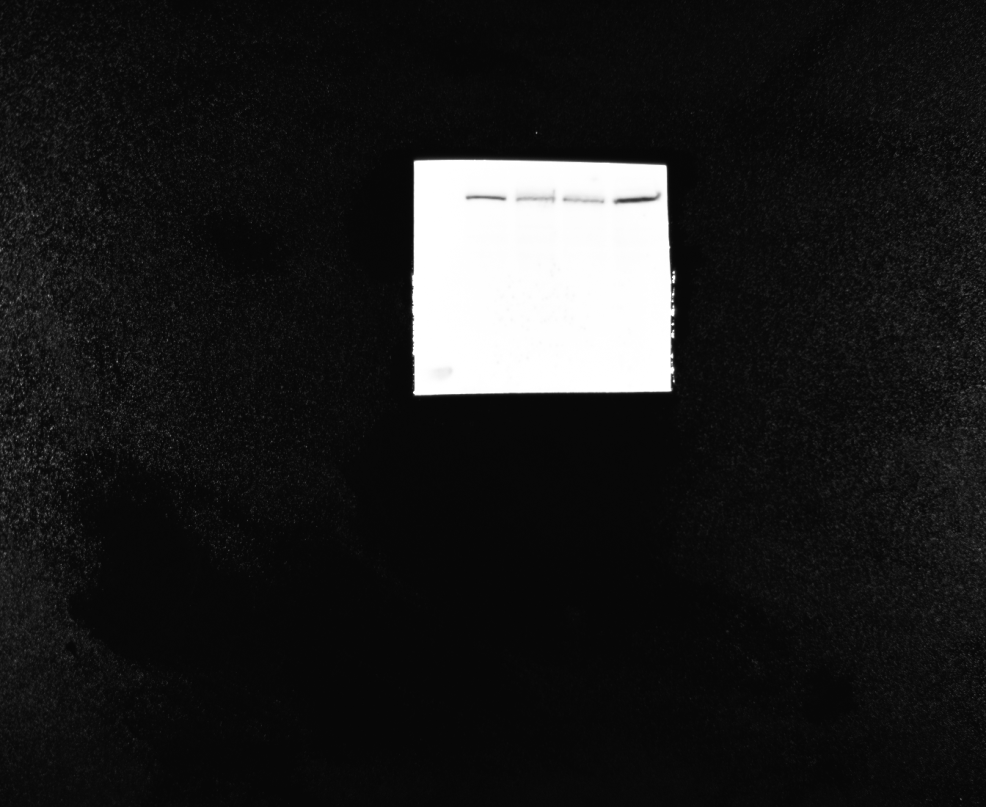


Figure 4B


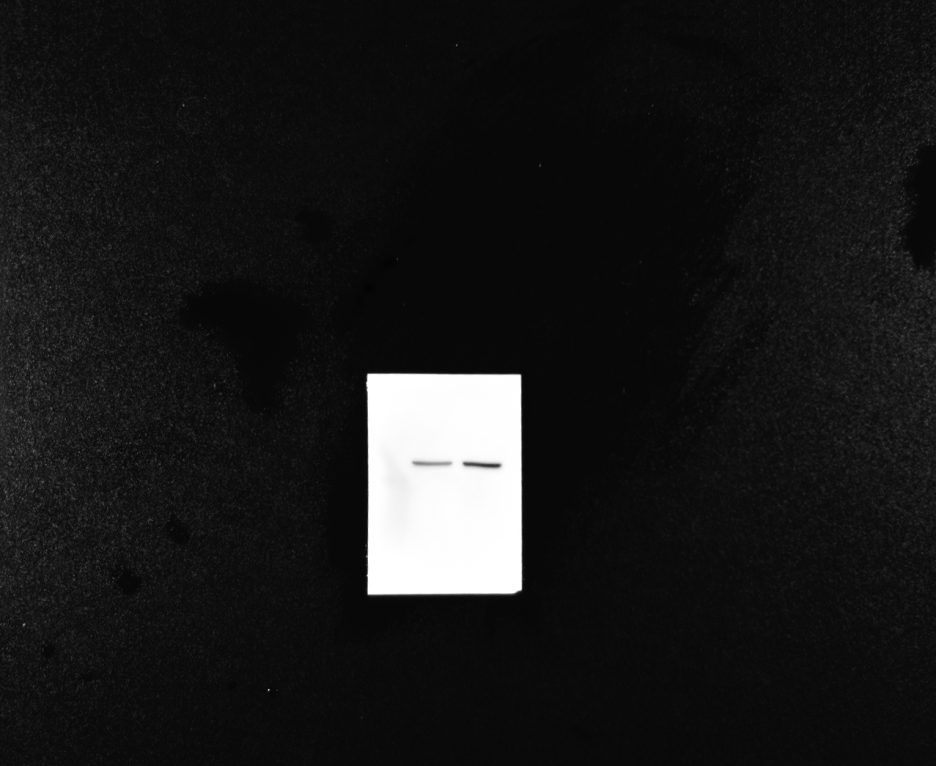

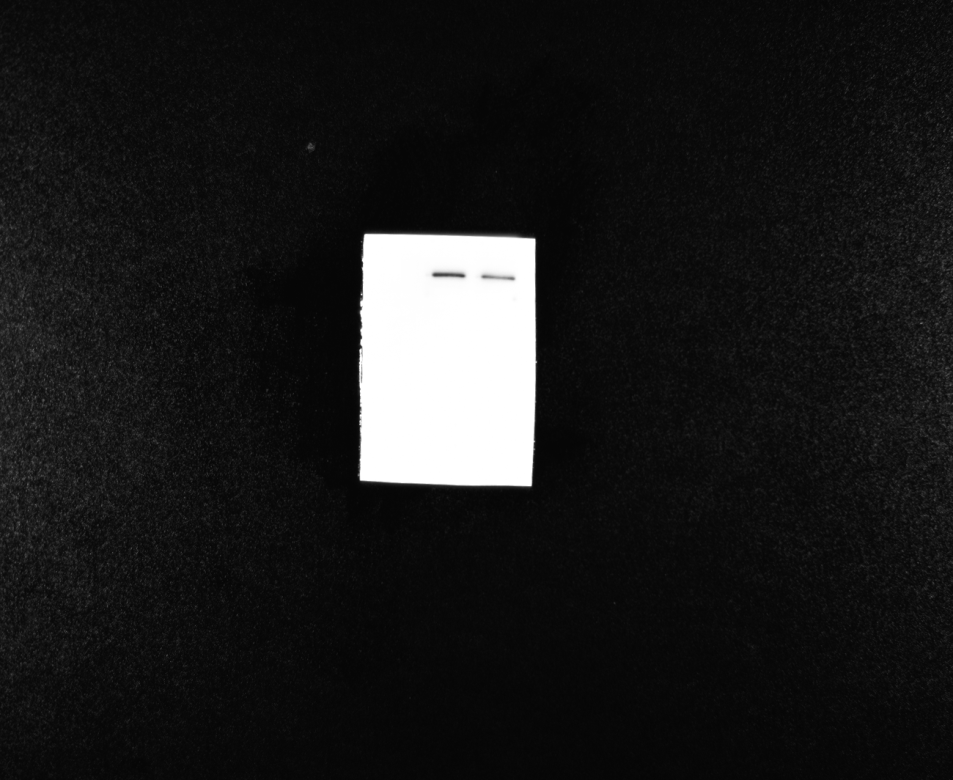

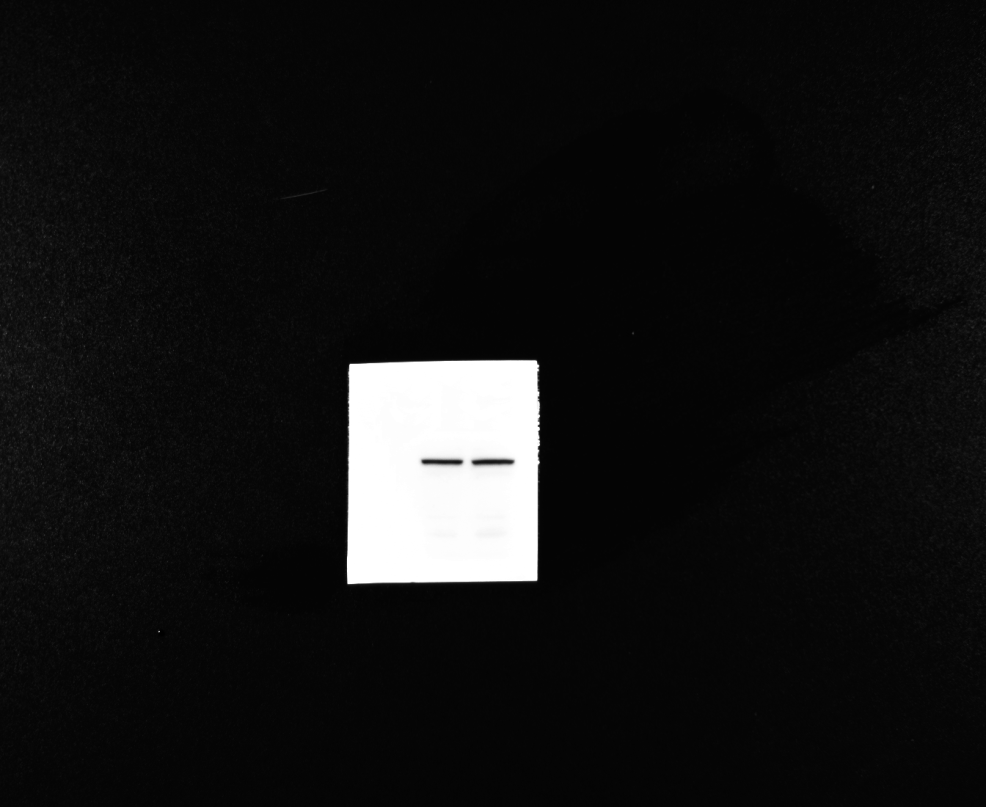


Figure 4H


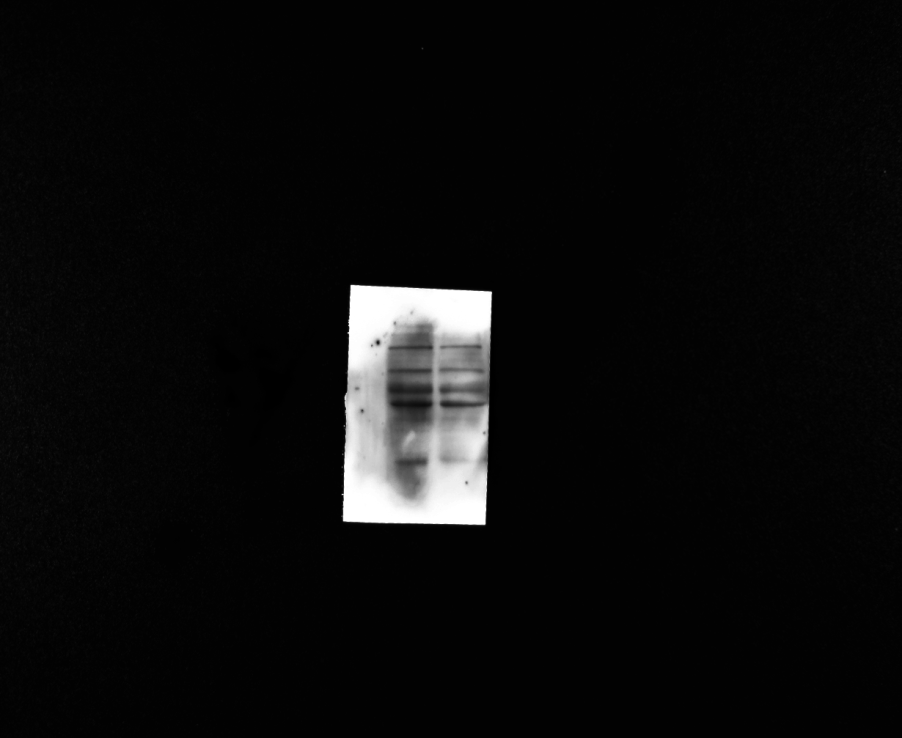

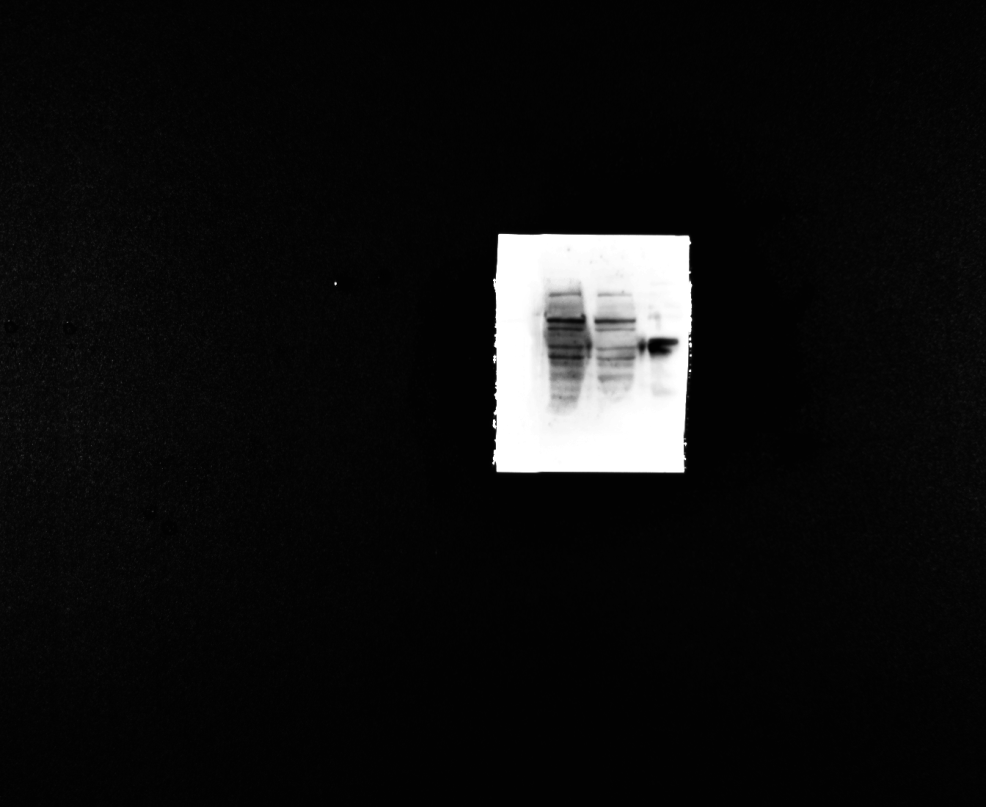

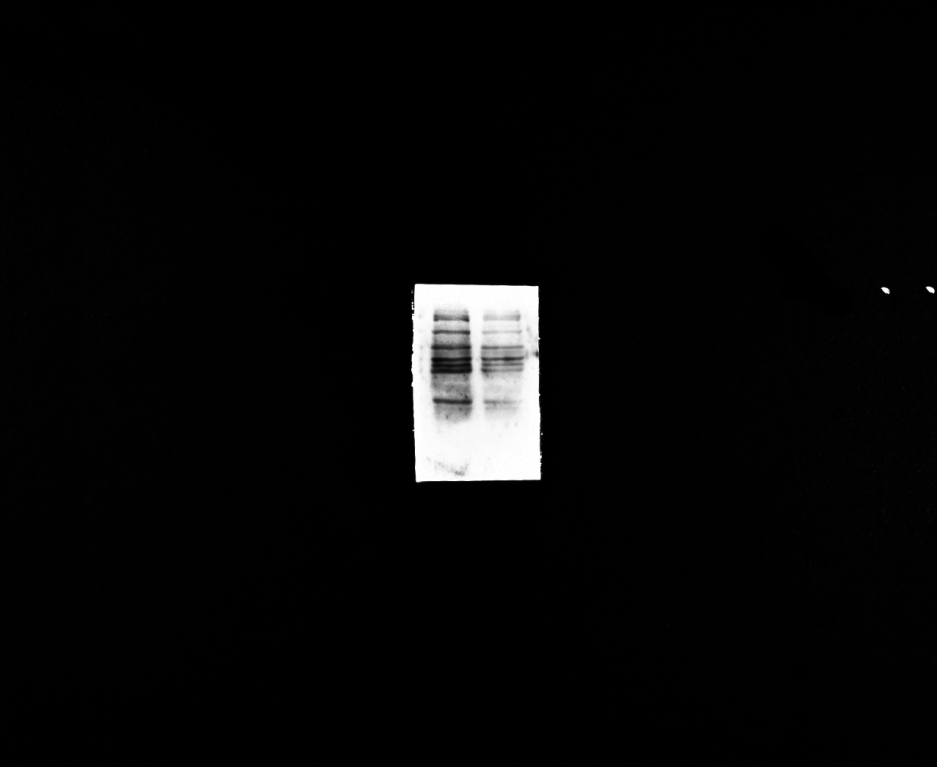

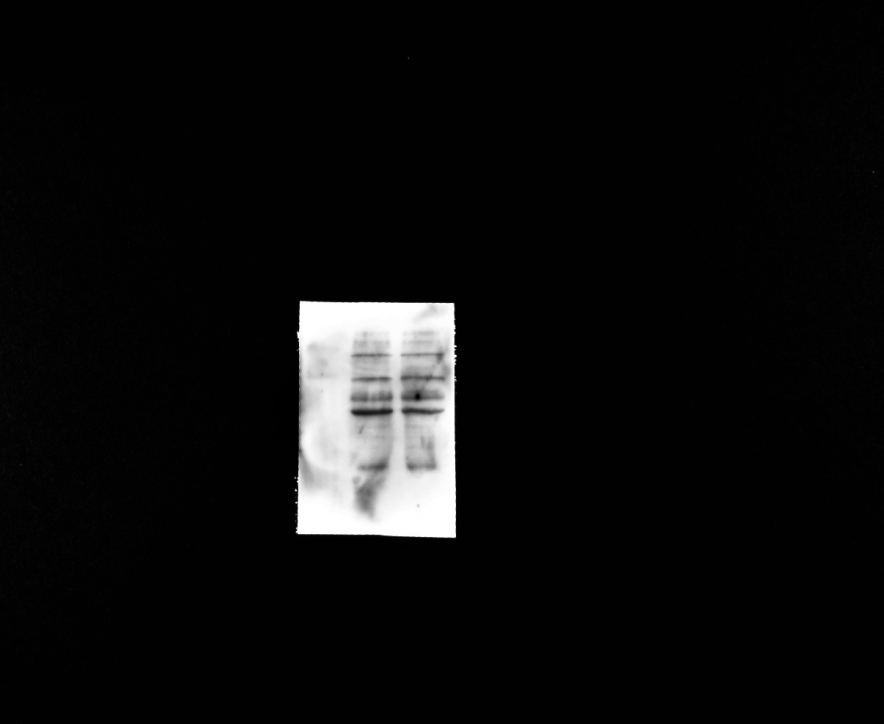

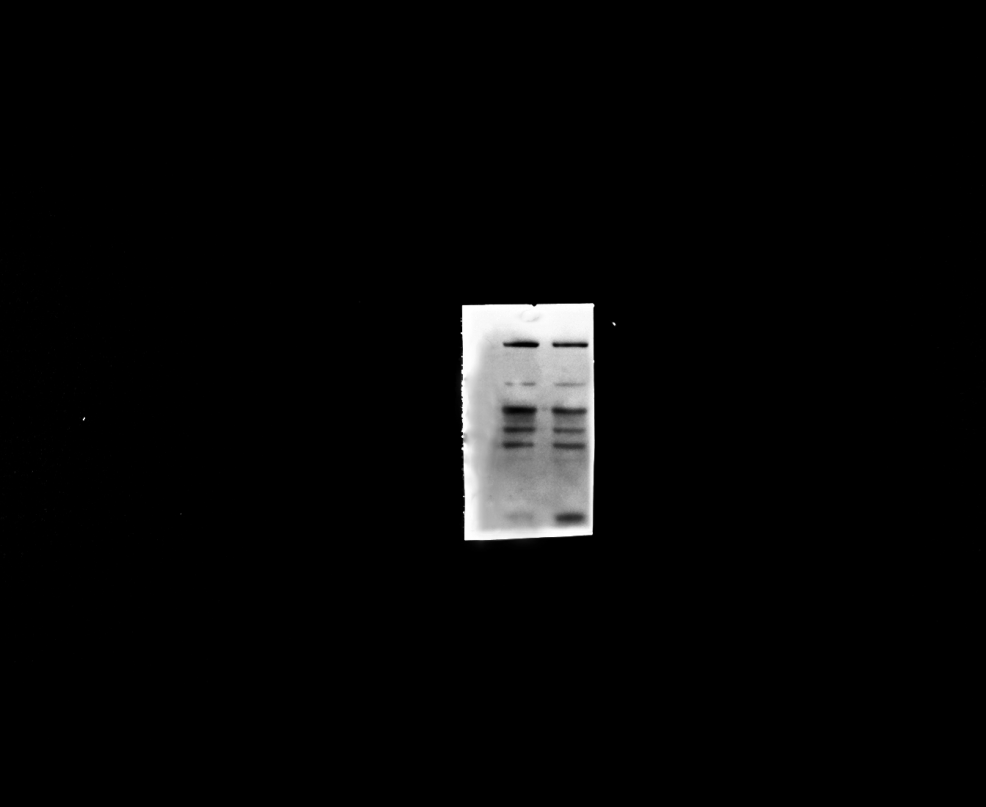


Figure 5B


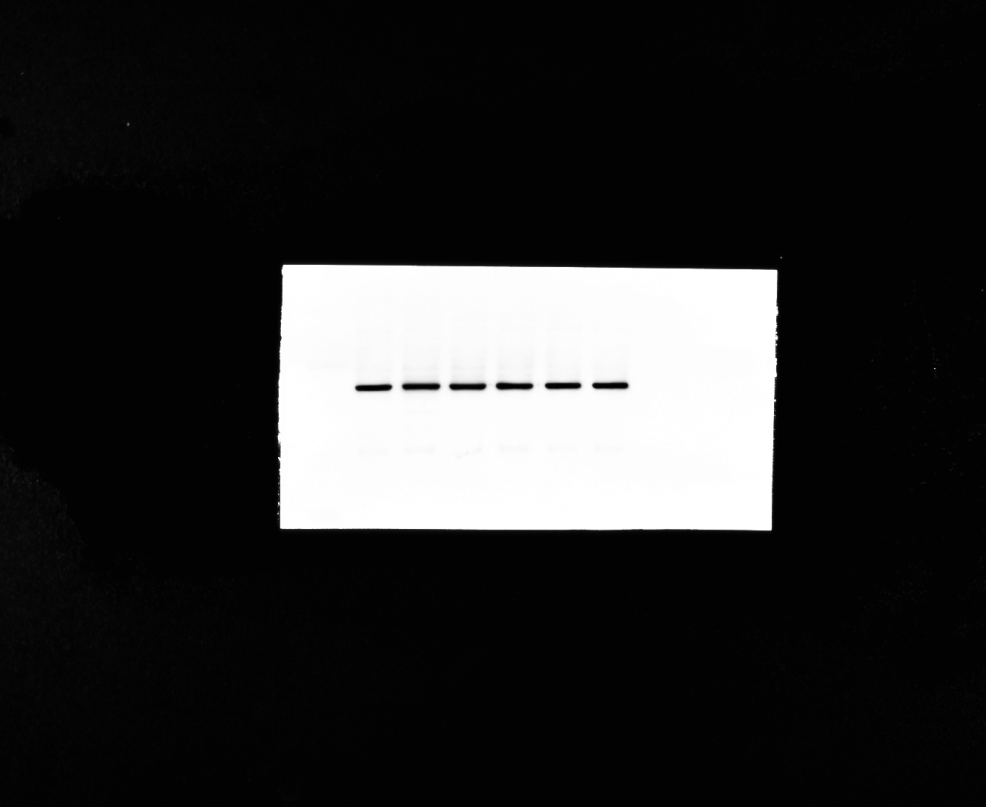

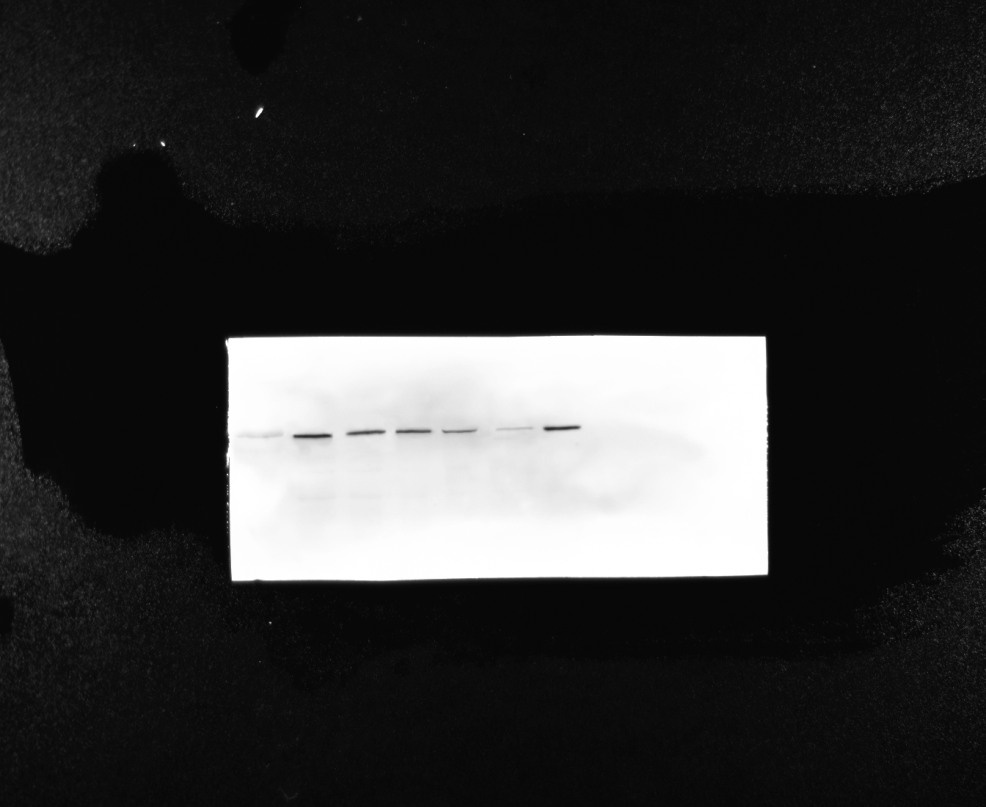

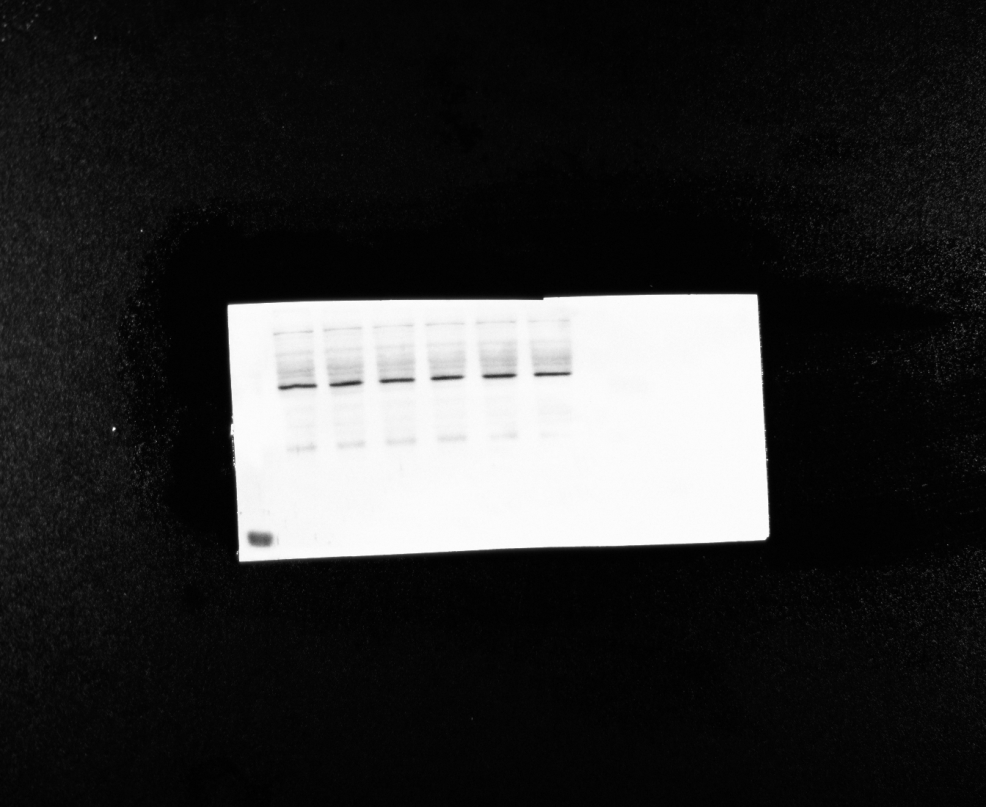


Figure 5C


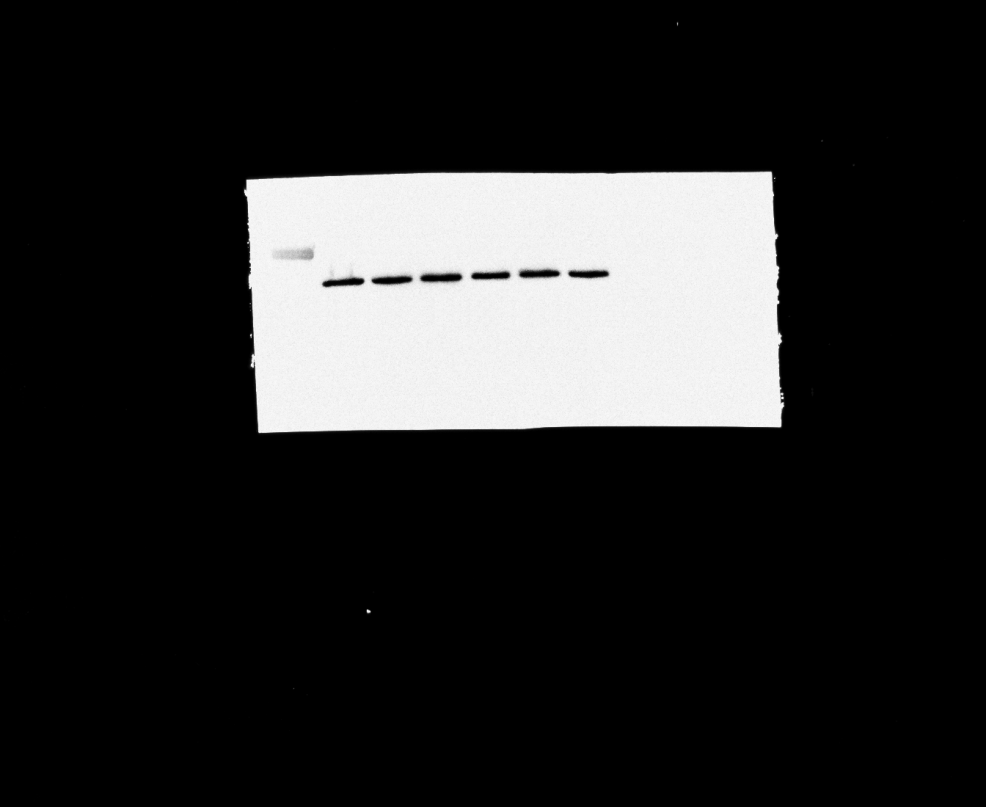

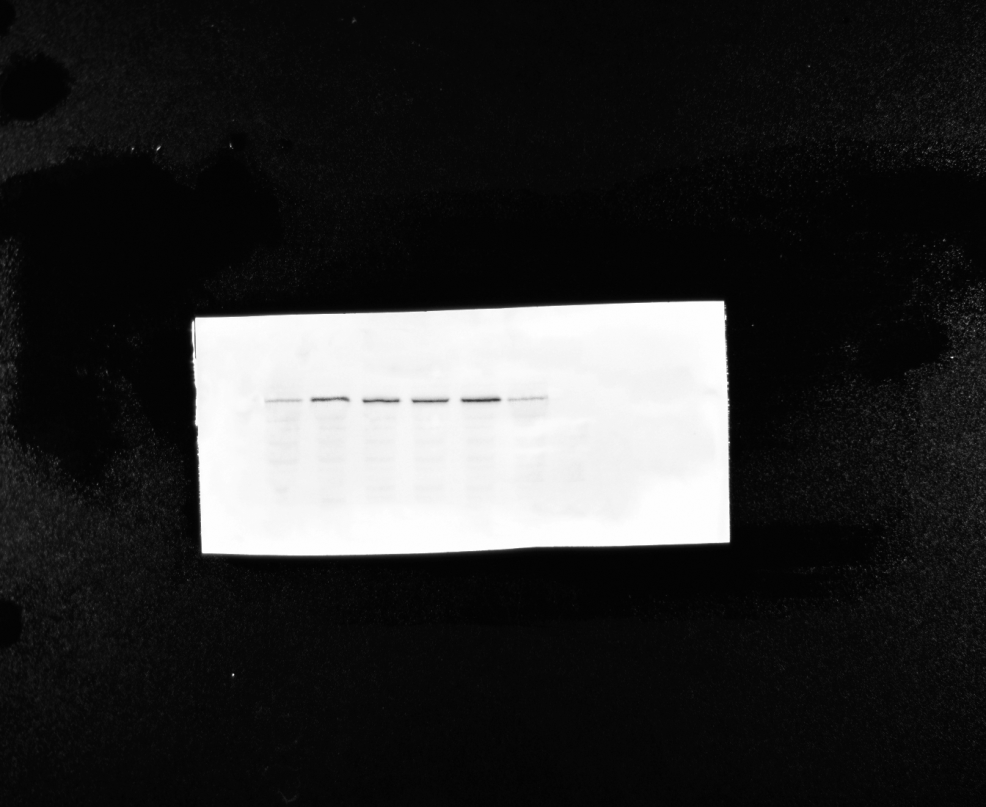

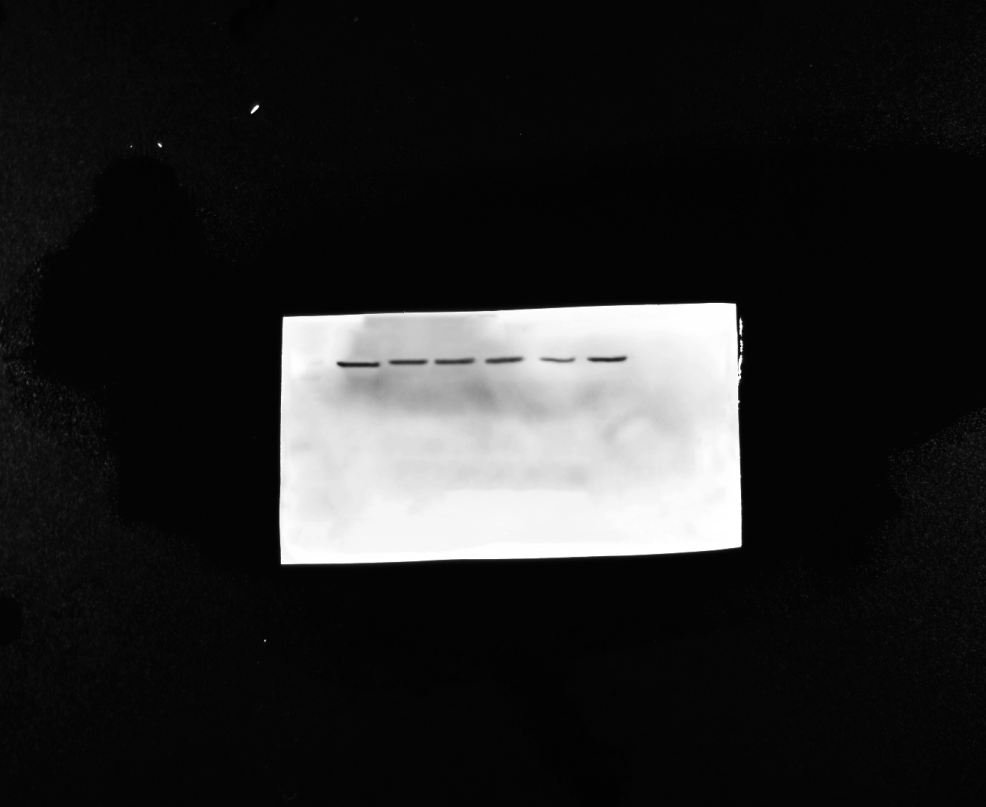

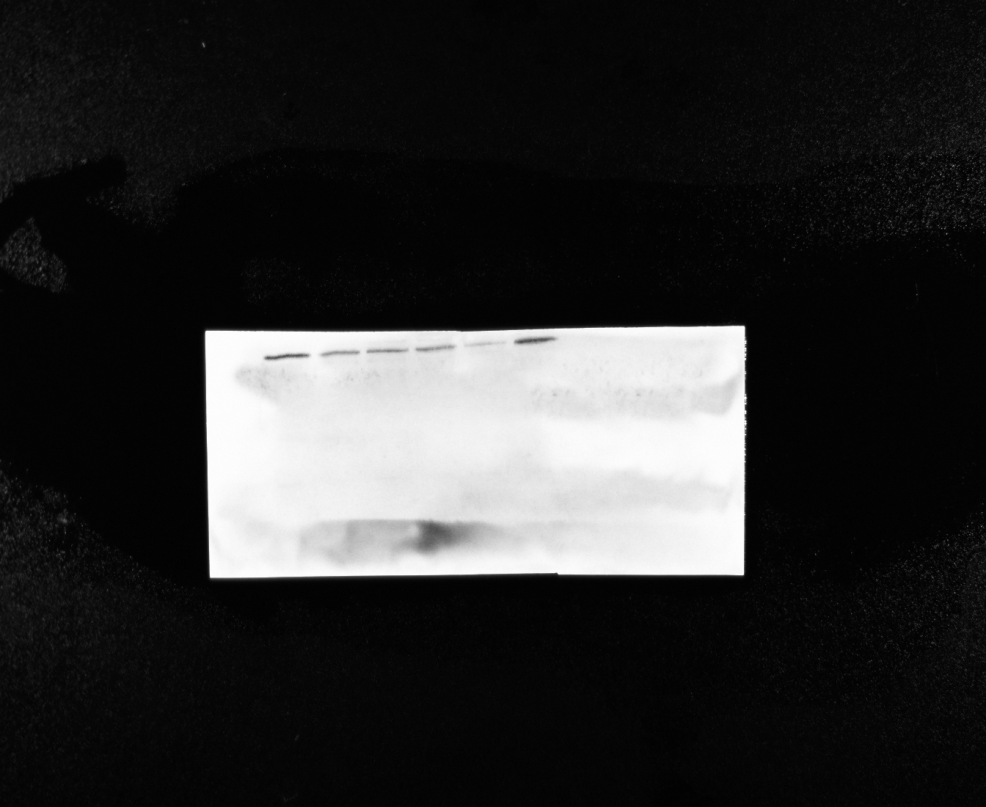


Figure 5E


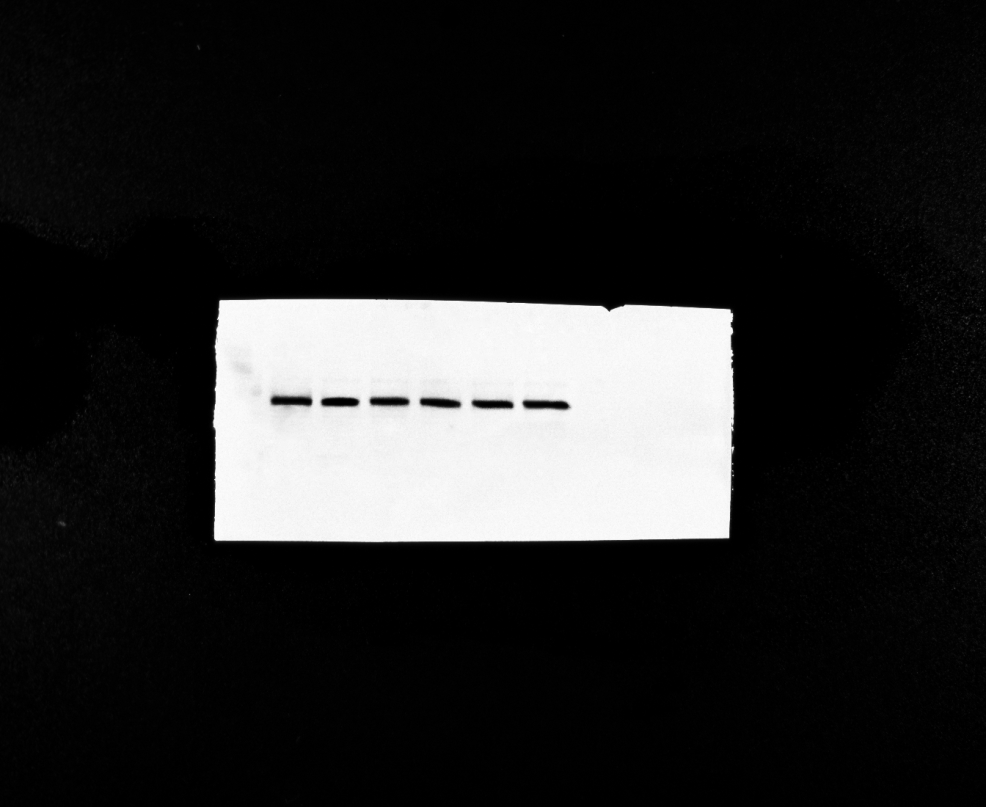

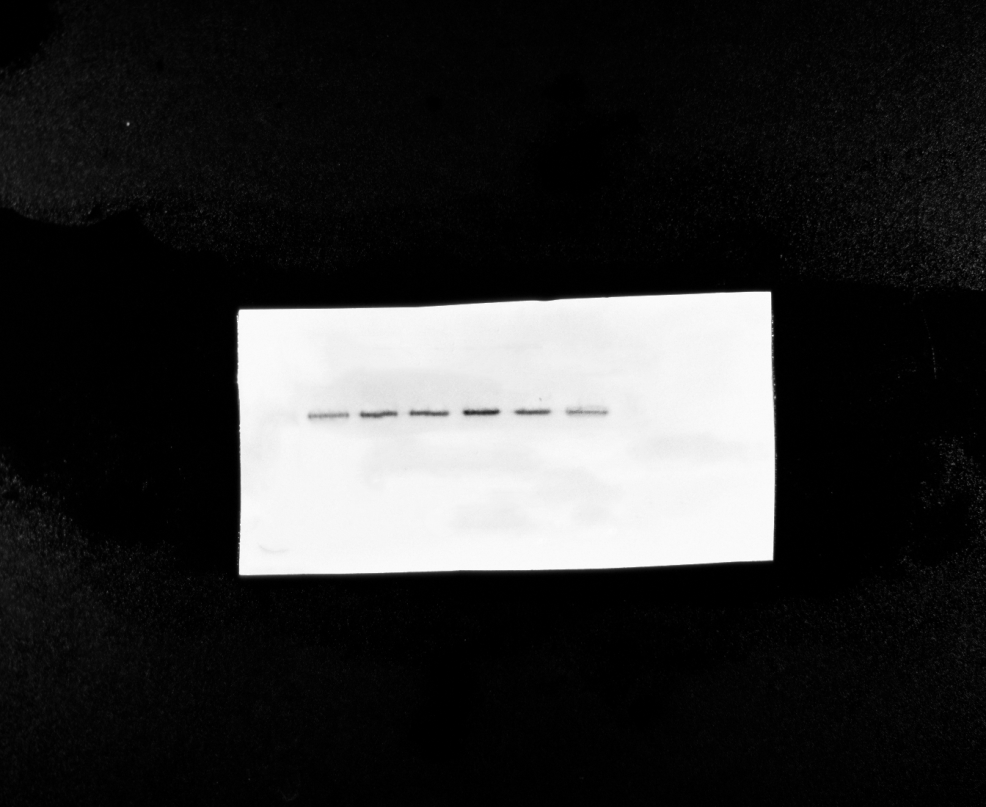

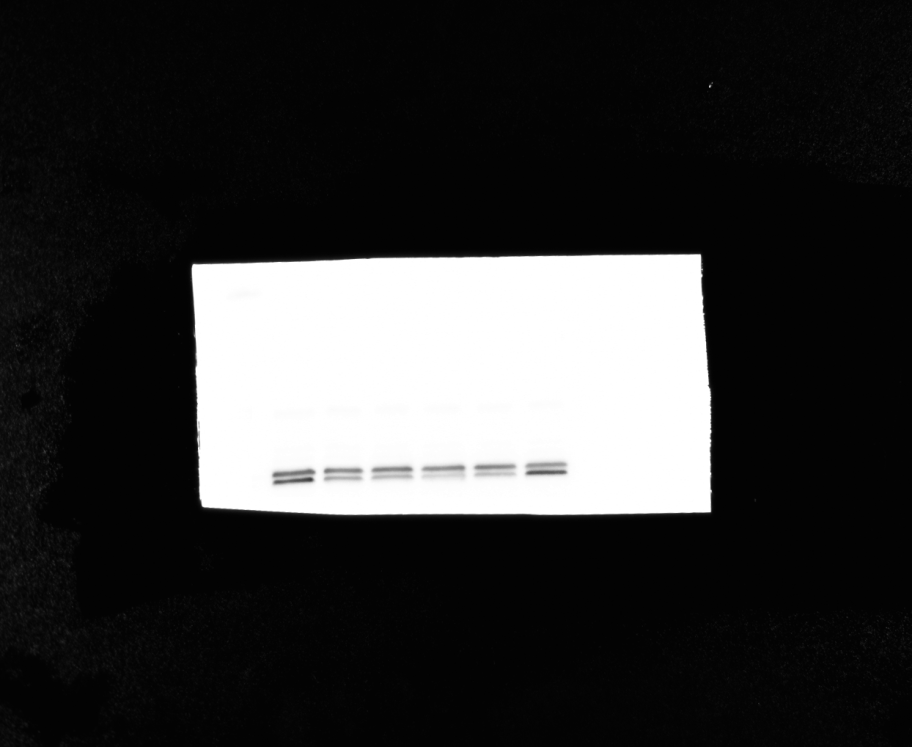

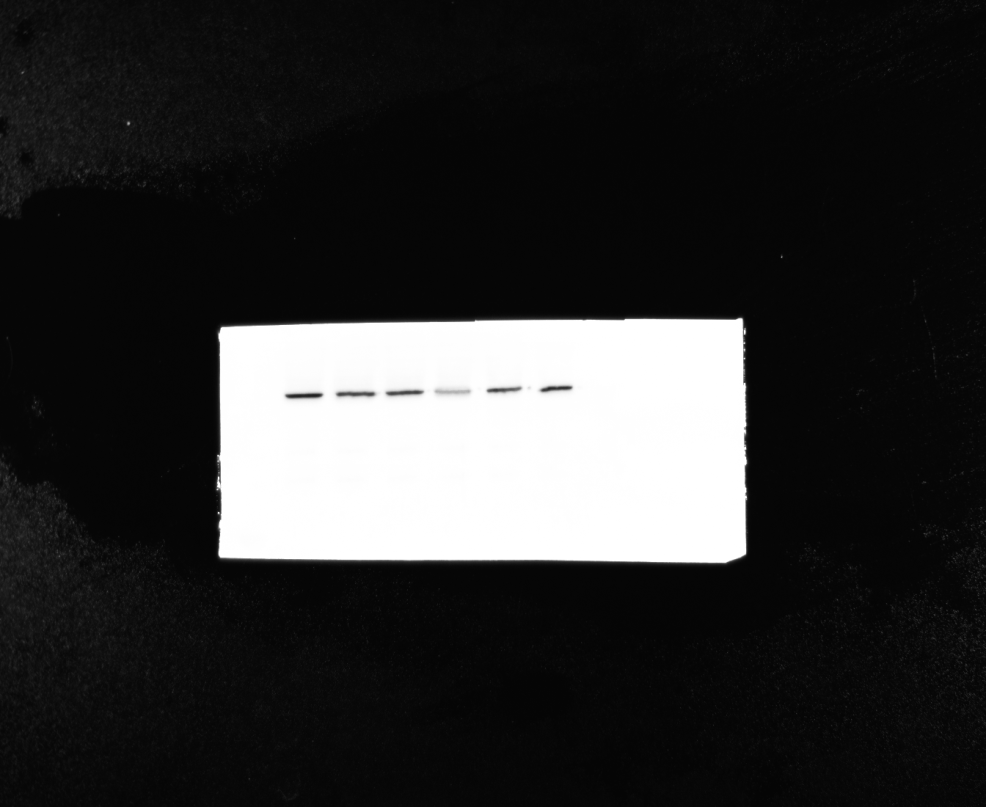


Figure 6B


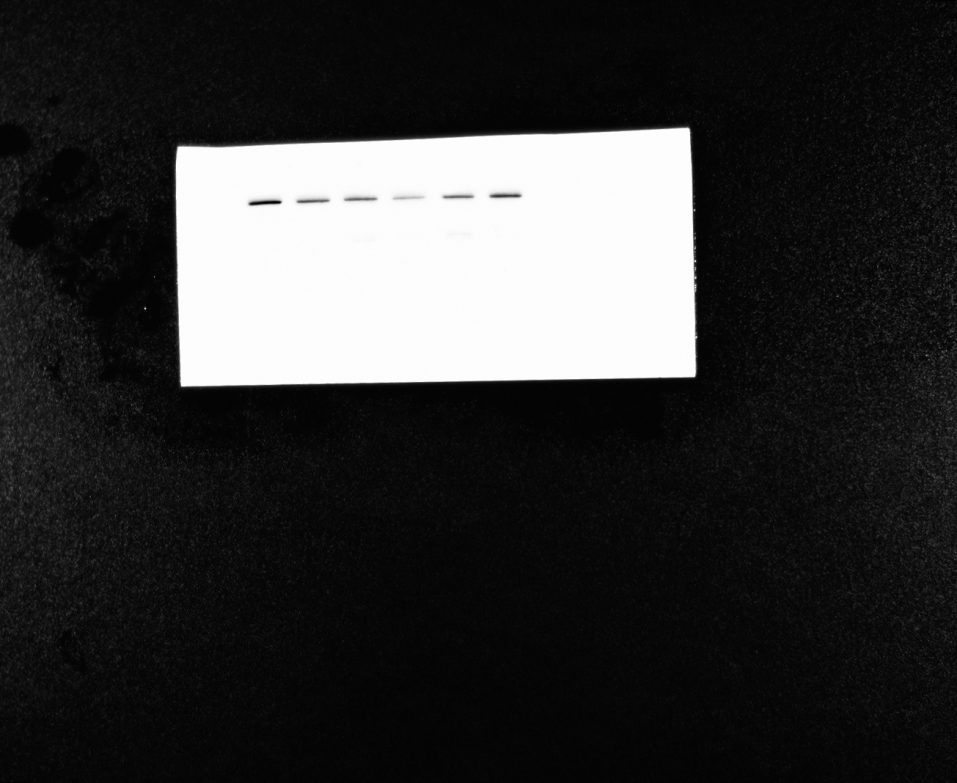

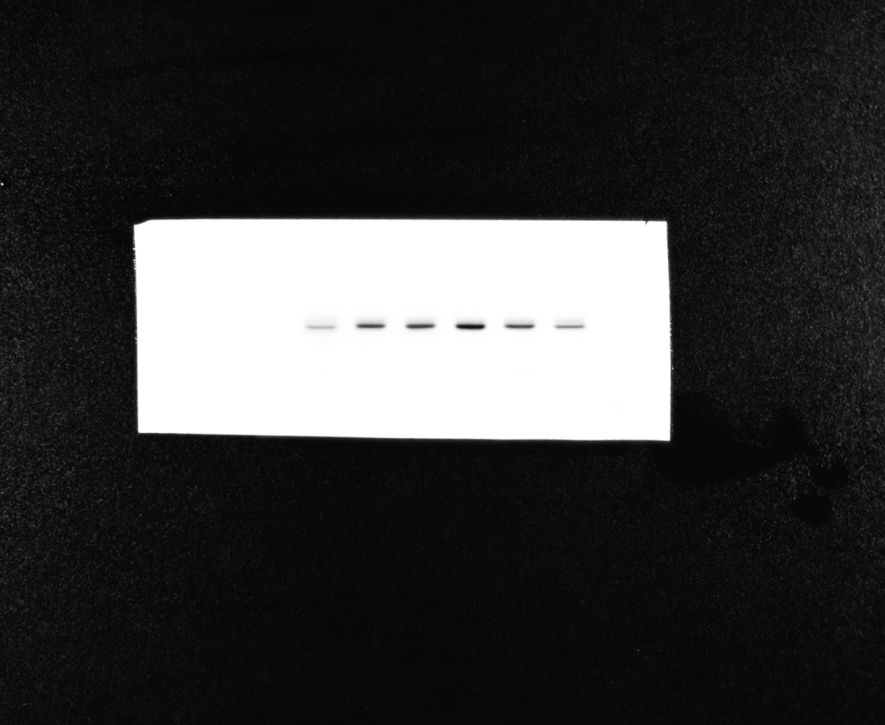

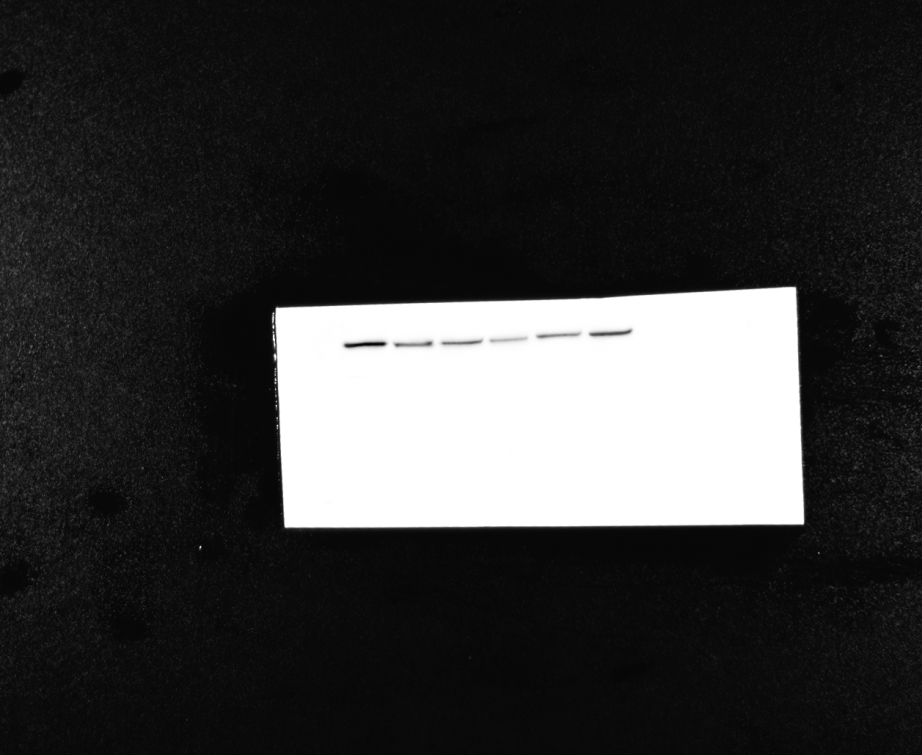

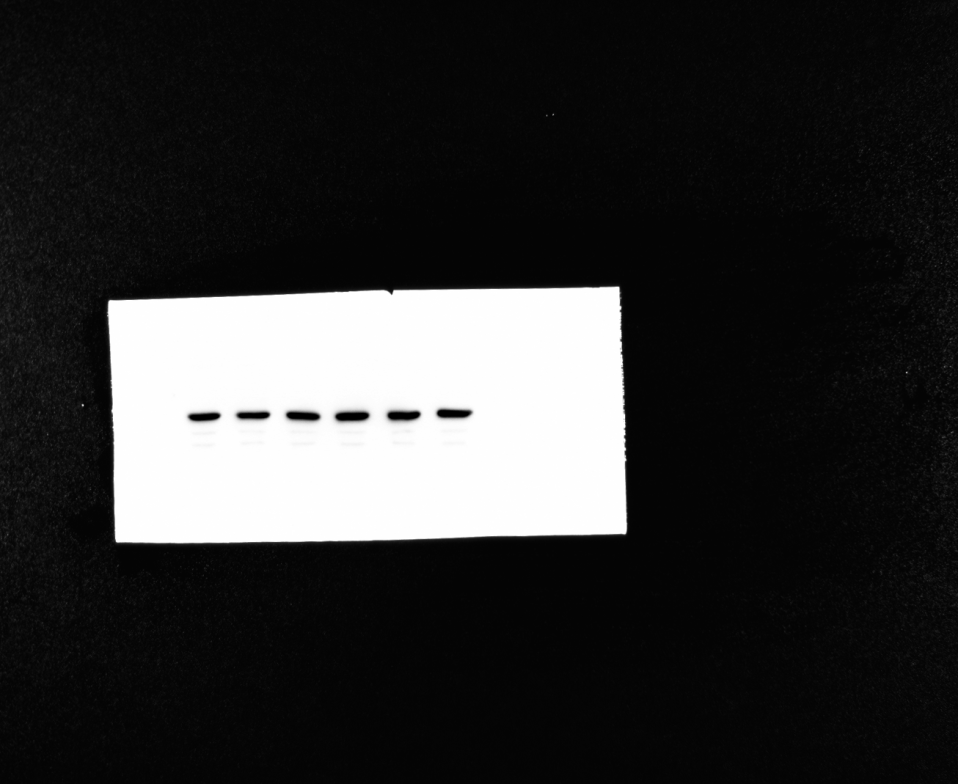


Figure 6C


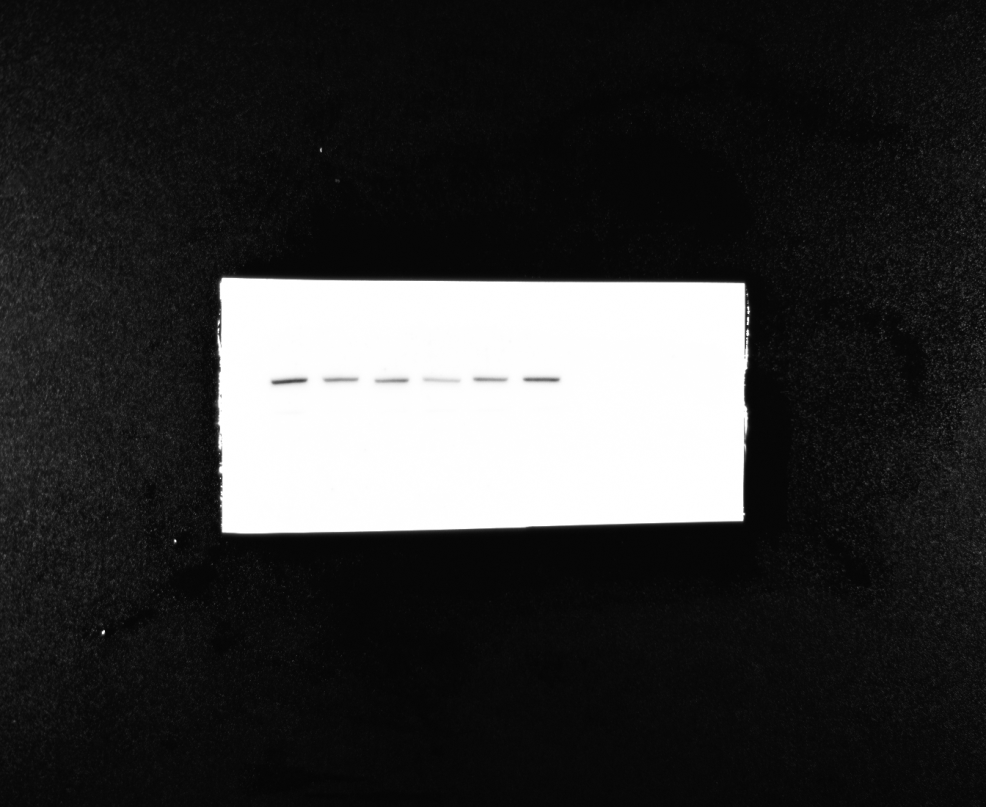

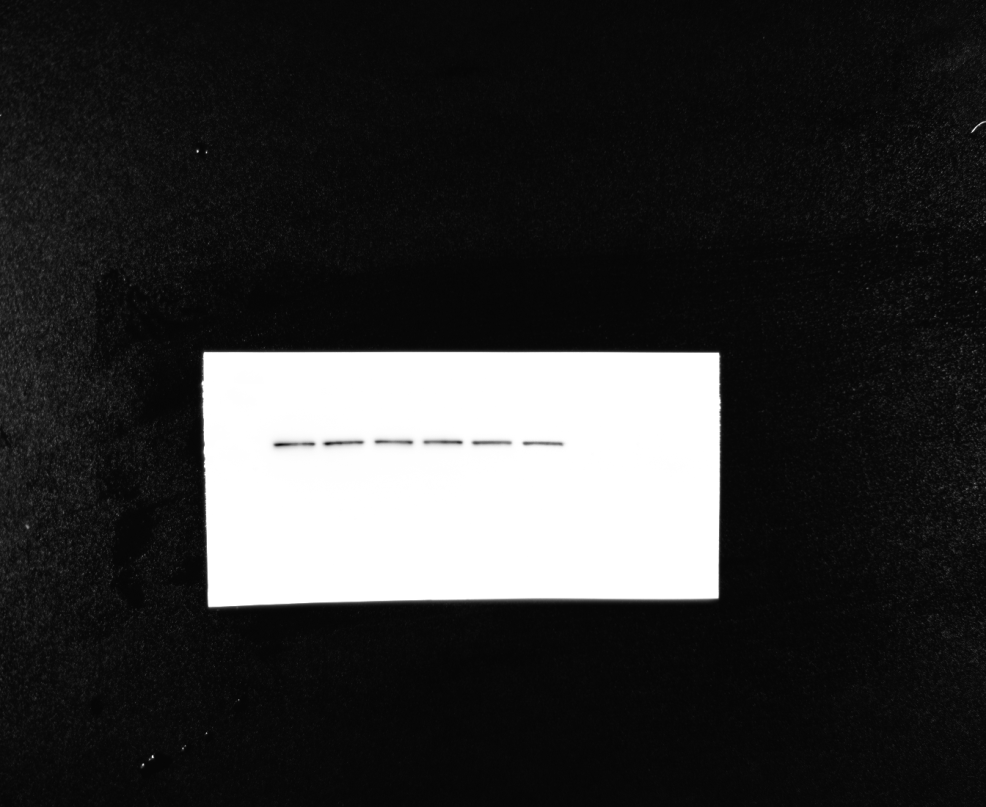

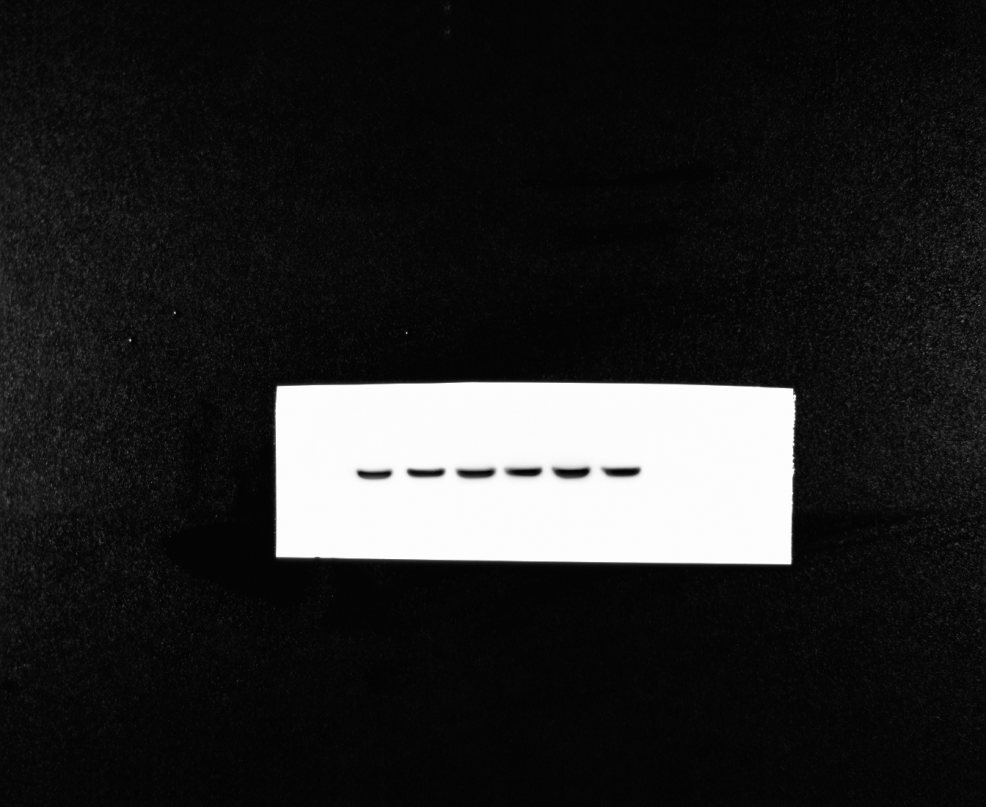


Figure 6D


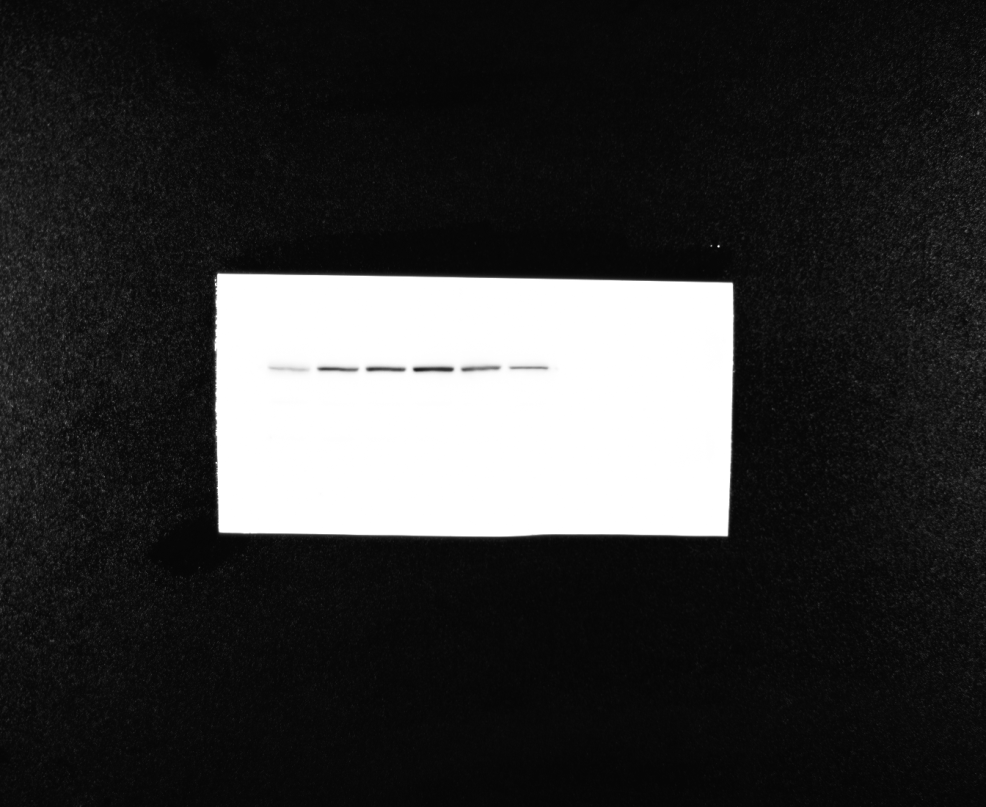

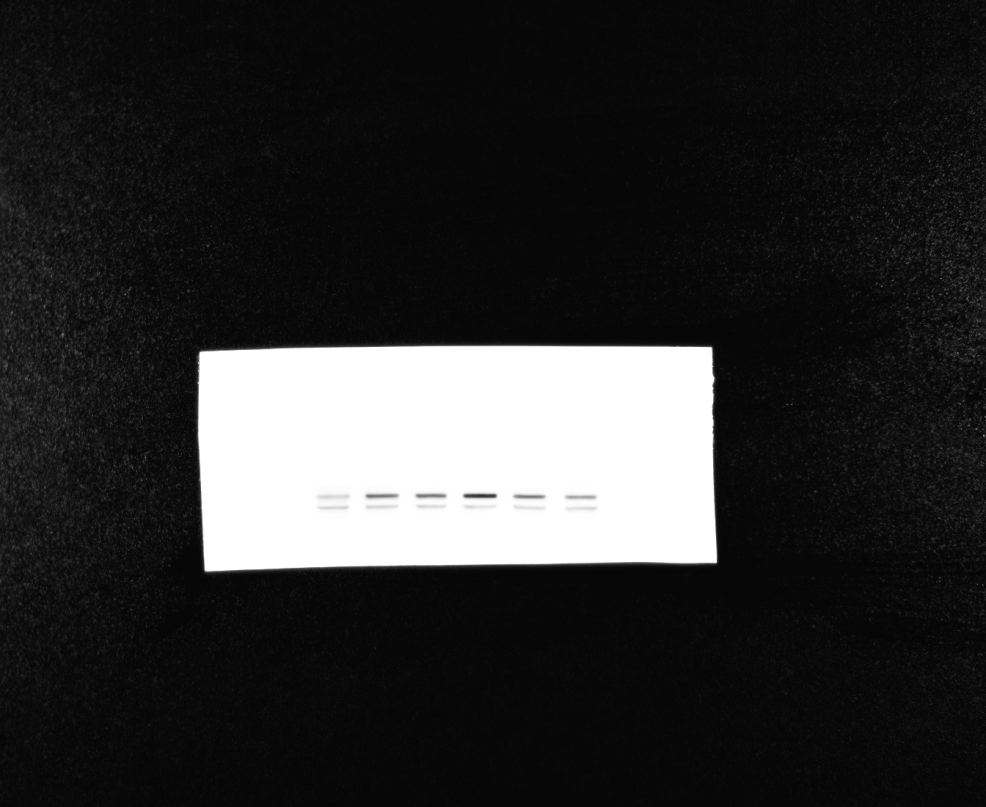

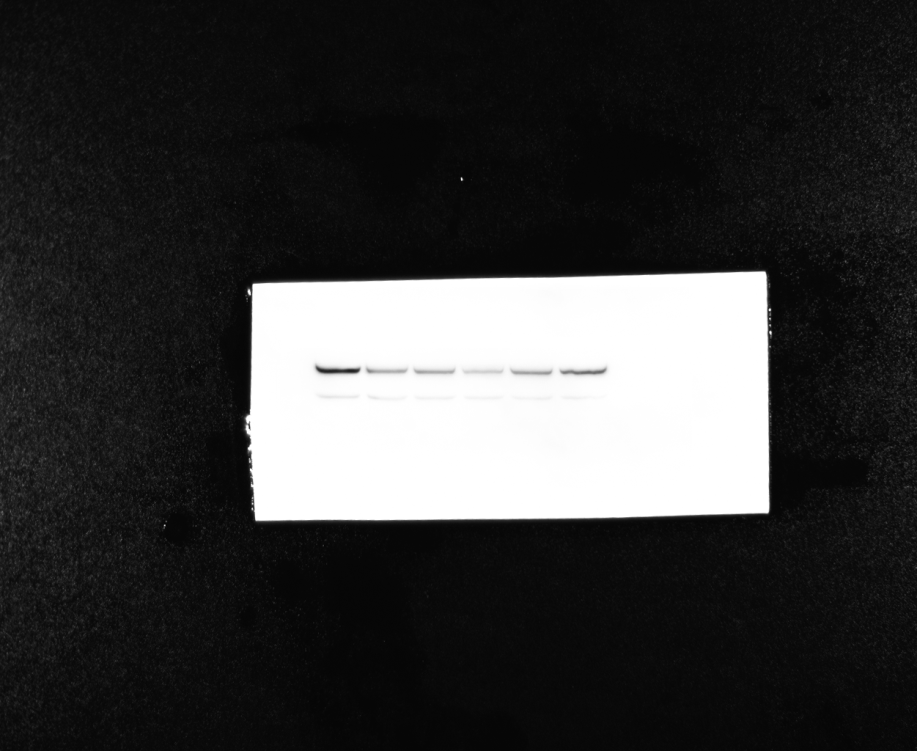

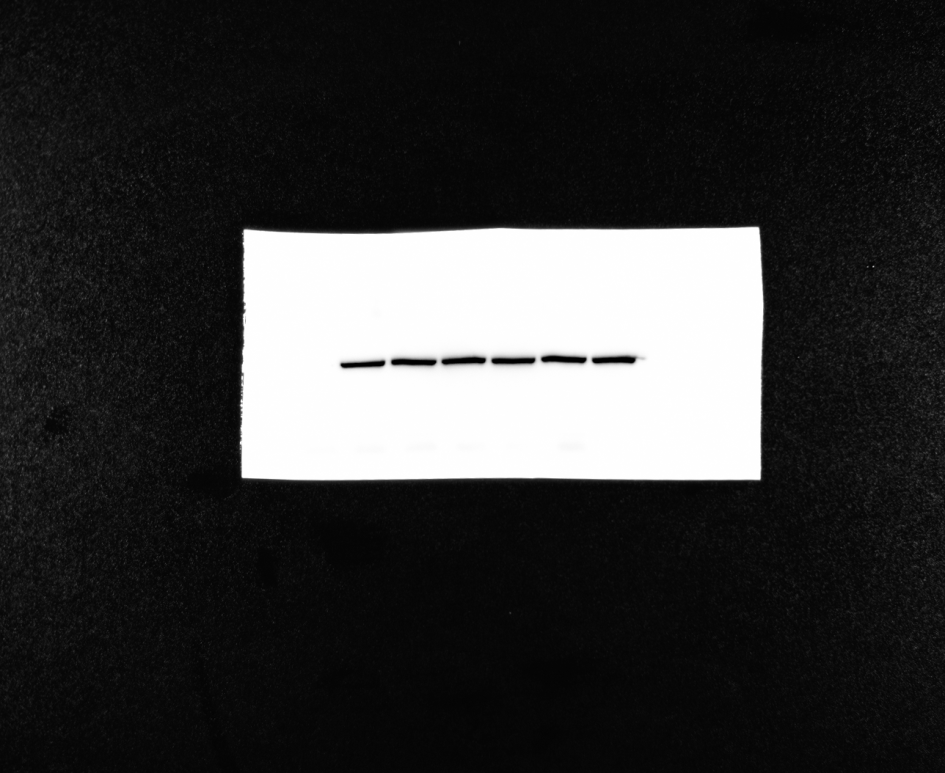


Figure 7D


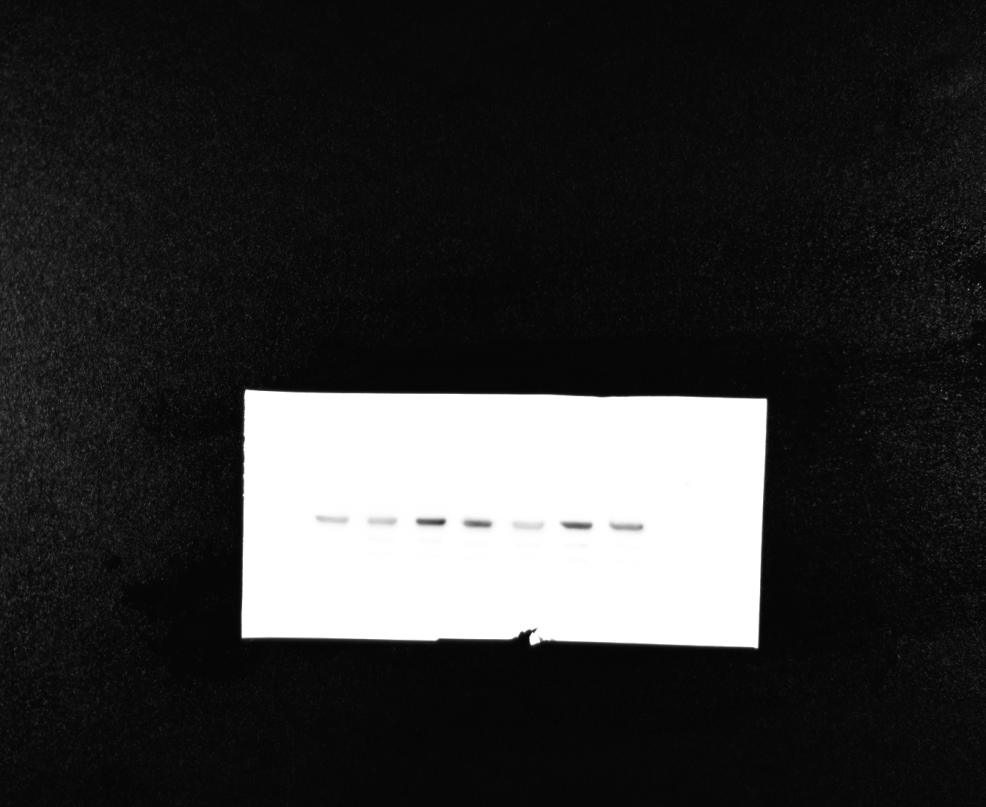

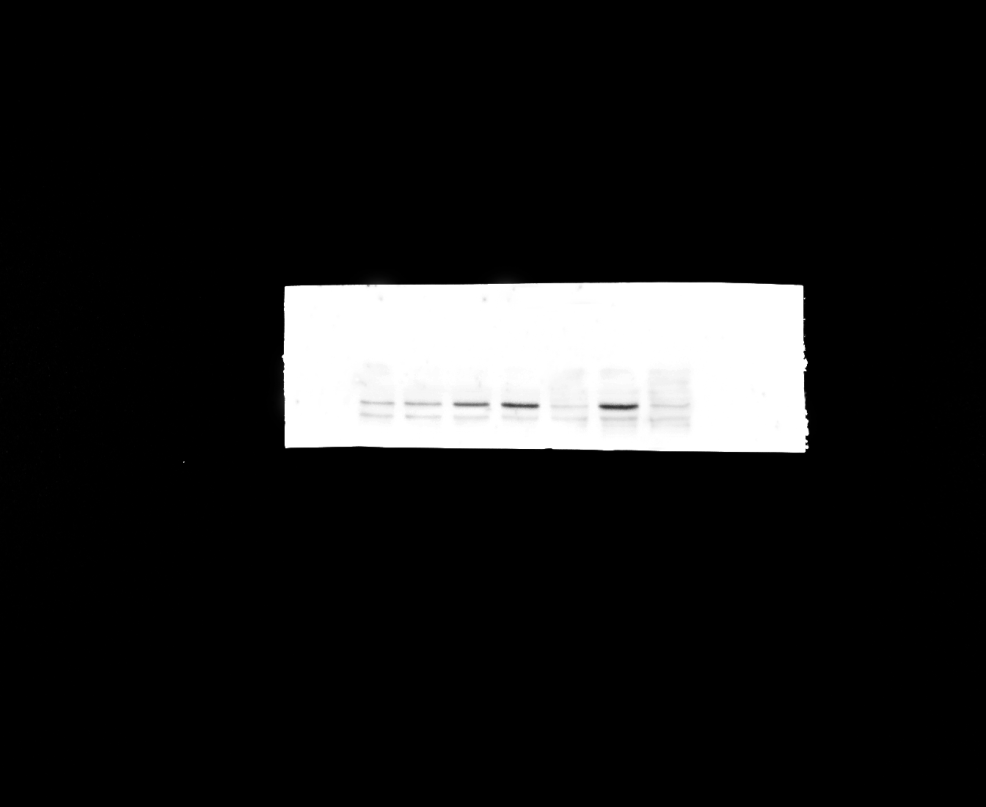

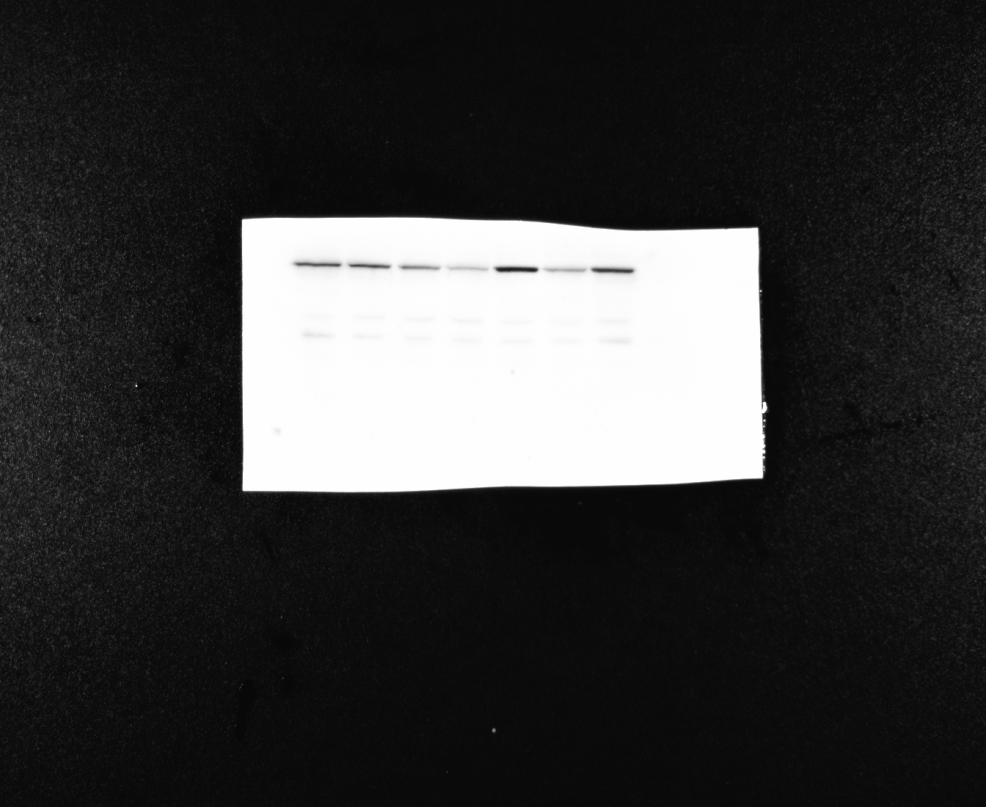

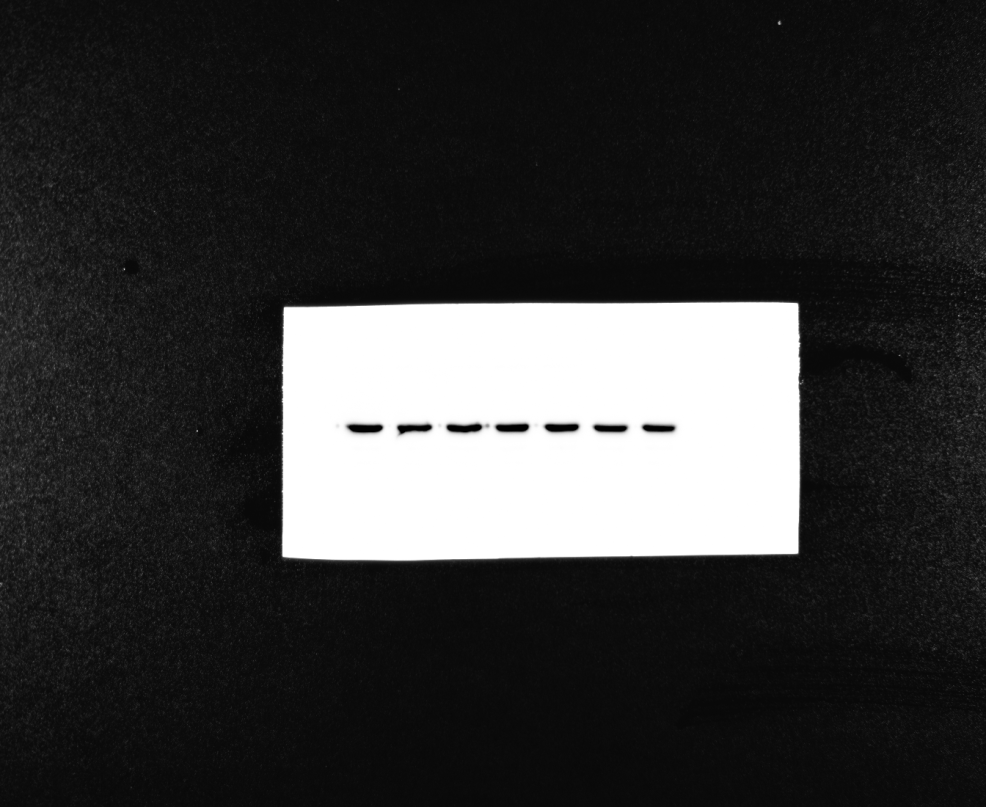

Supplement: Supplementary file 3 — uncropped western blots [file 41420_2022_896_MOESM3_ESM.docx]
